# High-throughput mitochondrial aerobic metabolism measurement: assessing a plate-reader approach using avian blood cells

**DOI:** 10.1242/bio.062712

**Published:** 2026-10-19

**Authors:** Cloe Hadjadji, Molly Ohse, Antoine Stier, Pierre Bize

**Affiliations:** ^1^ Swiss Ornithological Institute, Sempach, Seerose 1, CH-6206, Switzerland; ^2^ Department of Ecology and Evolution, University of Lausanne, Lausanne CH-1015, Switzerland; ^3^ Université de Strasbourg, CNRS, IPHC UMR 7178, F-67000 Strasbourg, France; ^4^ Department of Biology, University of Turku, Turku, FI-20014, Finland

**Keywords:** Plate reader, High-resolution respirometry, Oxidative phosphorylation, Proton leak, Bird

## Abstract

Mitochondria power biological processes, driving interest in measuring mitochondrial aerobic metabolism to understand individual variation in performance and fitness. High-resolution respirometry (HRR) is the gold standard because of its sensitivity and versatility, but it is time-consuming and has low throughput, limiting its use in large-scale studies. We assessed the validity of a high-throughput, fluorescence-based plate reader by comparing it to HRR. In a first experiment, we measured mitochondrial respiration in blood cells from two bird species (quails and chickens) across three temperatures (hypothermia, normothermia, and hyperthermia). The two methods were moderately correlated and revealed similar species effects; however, temperature effects differed between approaches, particularly under hyperthermic conditions, indicating that the plate-reader approach is best suited for stable, normothermic conditions. In a second experiment on sex differences in quails at normothermia, the two methods agreed quantitatively for proton leak and oxidative control efficiency and qualitatively for oxidative phosphorylation, although the plate-reader estimate was marginally significant (*P*=0.060 versus *P*=0.046 for HRR). The plate reader's main limitation is its lower sensitivity and greater measurement error, which could be offset by increasing sample sizes. Following protocol optimisation for each study system, this approach may offer a useful high-throughput complement to HRR for studies prioritising larger sample sizes.

## Teaser

**Summary:** A high-throughput plate-reader assay for mitochondrial respiration gives findings comparable to gold-standard high-resolution respirometry under normothermic conditions, offering parallel measurement of many samples at the cost of lower sensitivity.

## INTRODUCTION

Mitochondria are essential organelles found in nearly all eukaryotic cells and are known as cellular powerhouses due to their crucial role in energy production, including ATP generation (Alberts et al., 2015; Nicholls and Ferguson, 2013). ATP serves as the energy currency not only of cells but also of tissues and whole organisms. Furthermore, besides energy production, mitochondria are also involved in calcium signalling (Rizzuto et al., 2012; Baughman et al., 2011), production of reactive oxygen species, cell growth and death regulation (Green and Reed, 1998; Wang and Youle, 2009), and heat generation (Divakaruni and Brand, 2011; Nicholls and Ferguson, 2013). These functions highlight the multifaceted importance of mitochondria from cellular physiology to whole-organism metabolism. Consequently, there is a growing interest in measuring mitochondrial aerobic metabolism to gain insights into the source of individual variations in performance, life-history trade-offs, and fitness (Hood et al., 2018; Koch et al., 2021; Metcalfe et al., 2023).

Mitochondria generate ATP via oxidative phosphorylation (OXPHOS) (Mitchell, 1961; Saraste, 1999; Voet and Voet, 2011; Zhao et al., 2019; Nolfi-Donegan et al., 2020). In this process, metabolic substrates are oxidised to provide electrons to the mitochondrial electron transport system (ETS), whereas ADP acts as the phosphate acceptor for ATP synthesis. Electrons from NADH enter the ETS at Complex I (NADH dehydrogenase), and those from FADH_2_ at Complex II (succinate dehydrogenase), both converging at coenzyme Q, which transfers electrons to Complex III (cytochrome bc_1_ complex). Cytochrome c then carries electrons to Complex IV (cytochrome c oxidase), where molecular oxygen serves as the final electron acceptor and is reduced to water. Electron transfer through Complexes I, III, and IV is coupled to proton pumping across the inner mitochondrial membrane, generating the proton motive force that drives ATP synthesis by ATP synthase. Some protons, however, re-enter the matrix independently of ATP production [i.e. proton leak (LEAK)], reducing OXPHOS efficiency but helping to moderate mitochondrial membrane potential and reactive oxygen species formation (Rolfe and Brown, 1997; Brand, 2000; Divakaruni and Brand, 2011). In experimental *in vitro* studies, inhibitors such as oligomycin (i.e. an inhibitor of ATP synthase) are used to assess the relative contribution of LEAK and OXPHOS processes to cellular respiration, and an inhibitor of Complex III (e.g. antimycin A) is also used to determine residual non-mitochondrial oxygen flux.

The measurement of mitochondrial oxygen consumption has historically relied on polarographic (Clark-type) oxygen electrodes, which detect dissolved oxygen via an amperometric reaction at a platinum cathode within a small measurement chamber (Clark, 1956; Estabrook, 1967). This approach requires comparatively minimal and inexpensive equipment, making it accessible even to laboratories without dedicated respirometry instrumentation. It can be combined with substrate-uncoupler-inhibitor titration (SUIT) protocols to sequentially assess multiple respiratory states (Silva and Oliveira, 2012), but it can only measure one sample at a time, and its comparatively low sensitivity typically requires a higher mitochondrial density or respiratory flux to obtain a reliable signal. Building on this polarographic principle, high-resolution respirometry (HRR) systems, such as the Oroboros Oxygraph-2k (O2k) (Oroboros Instruments, Innsbruck, Austria), have become the gold standard for mitochondrial analysis due to their greater precision and sensitivity, which allow SUIT protocols to be applied reliably across a broader range of sample types and respiratory fluxes (Pesta and Gnaiger, 2012; Kuznetsov et al., 2008; Stier et al., 2017). This method also directly monitors oxygen consumption rates in real time within sealed chambers. Substrates and inhibitors are sequentially added to isolated mitochondria and intact or permeabilised cells, allowing precise, absolute quantification of respirations linked to OXPHOS and LEAK by tracking changes in oxygen concentration (Stier et al., 2017). However, like Clark-type electrodes, HRR systems retain a low-throughput format: the Oroboros O2k has only two chambers and requires approximately 2 h per run, notably due to long washing steps required to remove inhibitors from the chambers. Thus, HRR is a highly sensitive but low-throughput approach, which limits its suitability for studies requiring large sample sizes.

Plate-based approaches provide a high-throughput alternative to Clark-type electrodes and HRR by enabling measurements of mitochondrial respiration to be taken in parallel across multiwell plates rather than sequentially in single chambers. One such option is the Agilent Seahorse XF analyser, a microplate-based system that measures oxygen consumption and extracellular acidification rates in real time (Wu et al., 2007; Ferrick et al., 2008). The Seahorse offers higher throughput than HRR and has been widely adopted in cell biology and biomedical research. A direct comparison with the Oroboros O2k in human fibroblasts revealed consistent results for LEAK and endogenous respirations under native cellular conditions (ROUTINE), though electron transfer capacity was underestimated by the Seahorse (Zdrazilova et al., 2022). However, like HRR instruments, the Seahorse is a specialised and expensive instrument, and its associated consumable costs are high. It is therefore primarily found in medical laboratories with a dedicated focus on cellular metabolism, and to the best of our knowledge, it has not yet been applied in non-model or wild animal systems, which are often studied under the limited financial resources typical of ecological and evolutionary research. The approach evaluated in the present study differs in a crucial respect as it instead relies on a standard fluorescence microplate reader, which is a common and versatile piece of equipment in biological laboratories, used routinely for a wide range of biochemical and cell-based assays.

The plate-reader methods utilise a fluorescent probe in conjunction with a microplate reader, detecting changes in extracellular oxygen concentration by measuring the fluorescence lifetime of the probe, which is decreased by oxygen (Diepart et al., 2010; Rosenstock et al., 2022). By adding substrates and inhibitors directly to the wells, OXPHOS and LEAK can be assessed in parallel across multiple samples. This approach is time effective and scalable to larger sample sizes (Diepart et al., 2010; Rosenstock et al., 2022). This method has been used in cell biology and biomedical research (Morten et al., 2019; Rosenstock et al., 2022) but, to the best of our knowledge, has not previously been directly validated against HRR outside these fields.

This study aims to assess the validity of a high-throughput plate-reader approach using a fluorescence-based assay to measure mitochondrial respiration rates by comparing this method with the gold standard of HRR. We used avian red blood cells (RBCs) for this methodological comparison since they possess functional mitochondria (Stier et al., 2013) that can be used to measure mitochondrial respiration rates from small and minimally invasive samples (Stier et al., 2017). Statistically significant correlations have been reported between RBC mitochondrial respiration and that of other tissues (e.g. Stier et al., 2017, 2022; Casagrande et al., 2023) and between RBC respiration and whole-organism metabolic rate (Malkoc et al., 2021; Casagrande et al., 2023; Thoral et al., 2024), as well as significant relationships with whole-organismal traits such as flight performance (Ton et al., 2025; Oefele et al., 2026). However, it is important to note that these relationships are often modest in magnitude and, in some cases, involve shallow slopes (i.e. comparatively small changes in RBC respiration corresponding to much larger changes in tissue or whole-organism metabolism), which likely limits the sensitivity of RBC-based assays to detect subtle biological effects. The present study uses avian blood cells primarily as a practical system for method comparison.

To ensure sufficient biological variation in mitochondrial respiration rates, we conducted two experiments. In Experiment 1, we measured the mitochondrial respiration rates of blood cells from two avian model species (i.e. chicken and Japanese quail), and each blood sample (and thus respiration rates) was measured at three different temperatures. Indeed, mitochondrial respiration rates vary markedly between species (e.g. Barbe et al., 2023) and are directly affected by temperature. With increasing temperature, OXPHOS respiration usually increases to a lesser extent and reaches an upper limit earlier than LEAK respiration, leading to a decrease in mitochondrial efficiency at high temperatures (Sokolova, 2023; Watson et al., 2026). In Experiment 2, we explored more subtle variation at the intraspecies level in quail by investigating how sex influences mitochondrial aerobic metabolism. Sex differences were expected due to the hormonal regulation of mitochondrial function and the differing energetic costs of reproduction between males and females (Wolff et al., 2016), though evidence in birds remains limited. Indeed, no effect was found in non-breeding king penguins (Stier et al., 2019), while sex-specific patterns have been linked to breeding or reproductive status in king penguins and quail (Cossin-Sevrin et al., 2025; Beccardi et al., 2025 preprint), including an effect on mitochondrial coupling efficiency (though not OXPHOS and LEAK), specifically in quail (Stier et al., 2022).

To test the usefulness and validity of a plate-based approach to measure mitochondrial aerobic metabolism in avian blood cells (Fig. 1), first, we report the technical repeatability of respiration measurements obtained with a plate reader. Indeed, a plate-based approach allows measurement of duplicates on the same microplate at the same time, whereas the HRR approach, when running samples in parallel, relies on different chambers and electrodes. Second, we tested whether the mitochondrial respiration rates measured in parallel in the same samples using both methods are correlated. Third, we assessed whether both approaches yield similar effect sizes and biological conclusions with a given experimental design, namely, testing differences in mitochondrial traits between species and at different assay temperatures, as well as within-species differences between sexes.

## RESULTS

### Technical repeatability of mitochondrial respiration measured with the plate reader

Repeatability is reported separately for each temperature, meaning that measurements at 40°C from Experiments 1 and 2 were combined. For OXPHOS_CI+CII_, repeatability of duplicate measurements was 0.94 (95% confidence interval (CI) [0.88, 0.98]; *n*=36) at 36°C, 0.98 (95% CI [0.96, 0.99]; *n*=59) at 40°C, and 0.86 (95% CI [0.66, 0.95]; *n*=36) at 44°C. For LEAK_CI+II_, repeatability of duplicate measurements was 0.72 (95% CI [0.30, 0.91]; *n*=28) at 36°C, 0.71 (95% CI [0.50, 0.85]; *n*=57) at 40°C, and 0.67 (95% CI [0.20, 0.86]; *n*=25) at 44°C. For oxidative control efficiency (OxCE), repeatability of duplicate calculations was 0.95 (95% CI [0.85, 0.98]; *n*=27) at 36°C, 0.25 (95% CI [0, 0.60]; *n*=59) at 40°C, and 0 (95% CI [0, 0.67]; *n*=23) at 44°C. Repeatability was therefore high for OXPHOS_CI+CII_ and LEAK_CI+II_ across all temperatures, but much lower and more uncertain for OxCE, particularly at 40°C and 44°C.

**Fig. 1.**
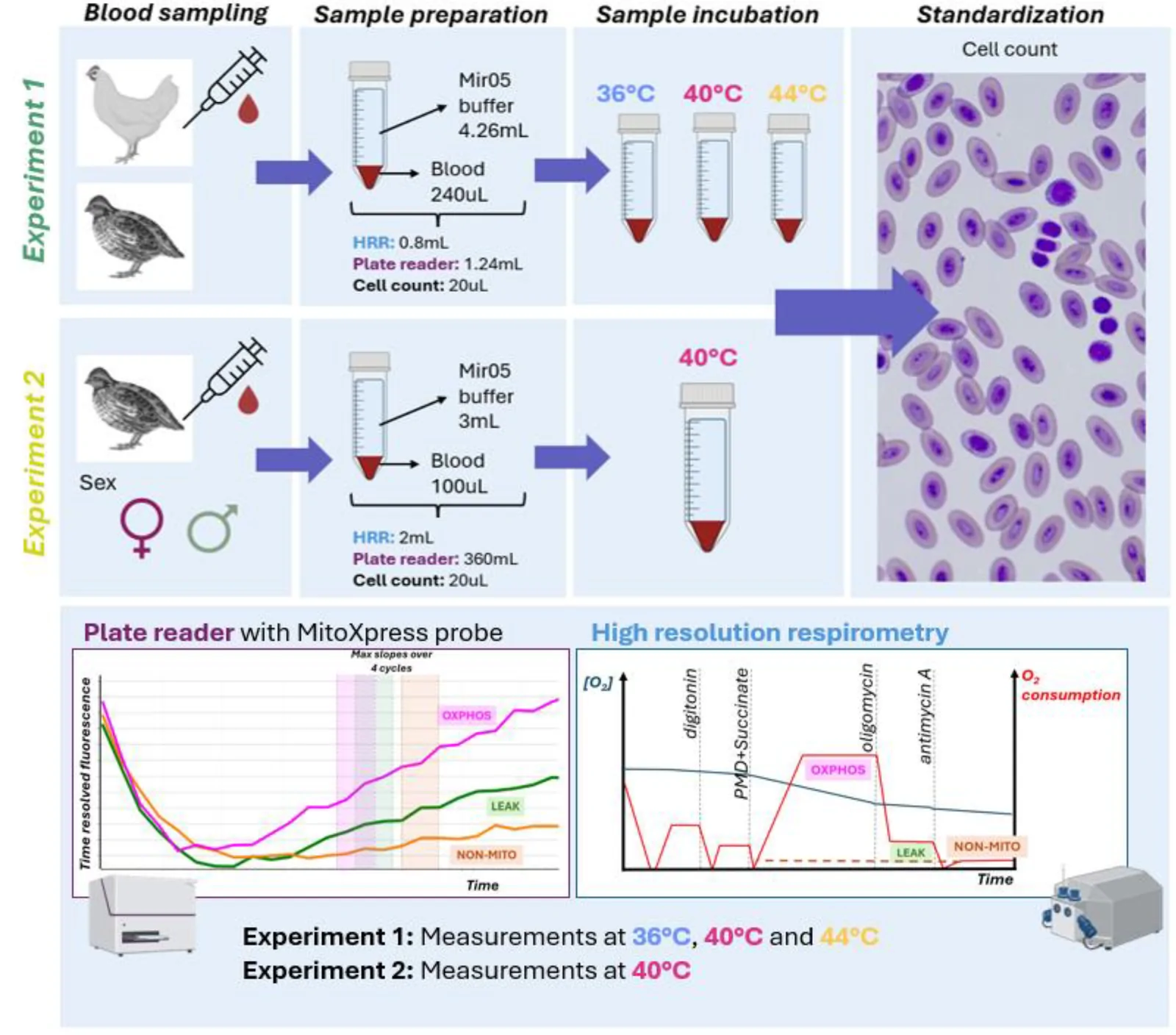
**Graphical summary of the two experimental designs performed in the present study aimed to compare measures of mitochondrial respiration rates using a plate-reader and fluorescent probe methods (i.e. VANTAstar microplate reader from BMG Labtech GmbH; Ortenberg, Germany) or an HRR method (i.e. O2k HRRs from Oroboros Instruments; Innsbruck, Austria).** *Experiment 1*: Whole blood in two species, Japanese quail and chicken, was measured at three different temperatures, using the two different methods. The aim was to evaluate species differences and to compare the responses of mitochondrial traits to *in vitro* temperature change in two species. *Experiment 2*: Whole blood in Japanese quail was measured at 40°C, using the two different methods to compare mitochondrial traits between sexes. Avian RBC image courtesy of Pia Cigler, Vetsuisse Faculty, University of Bern.

### Experiment 1: Species and temperature comparison

#### Correlations between the plate-reader and HRR methods

Measurements of mitochondrial traits obtained with the plate-based method were positively correlated with those obtained using the HRR method (Fig. 2). Pearson's correlation coefficients were 0.60 (95% CI [0.38, 0.76]; *n*=47) for OXPHOS_CI+CII_, 0.64 (95% CI [0.38, 0.81]; *n*=38) for LEAK_CI+CII_, and 0.42 (95% CI [0.02, 0.70]; *n*=24) for OxCE. These correlations were statistically significant (95% CI excluding zero) for all three traits. When stratified by temperature (and thus using smaller sample sizes), Pearson's correlation coefficients ranged from 0.53 to 0.88 for OXPHOS, from −0.07 to 0.74 for LEAK, and from 0.26 to 0.99 for OxCE. A detailed table of coefficients and an additional figure are presented in the supplementary materials for each temperature, species (altogether and separated) and mitochondrial traits (Table S4, Fig. S3).

**Fig. 2.**
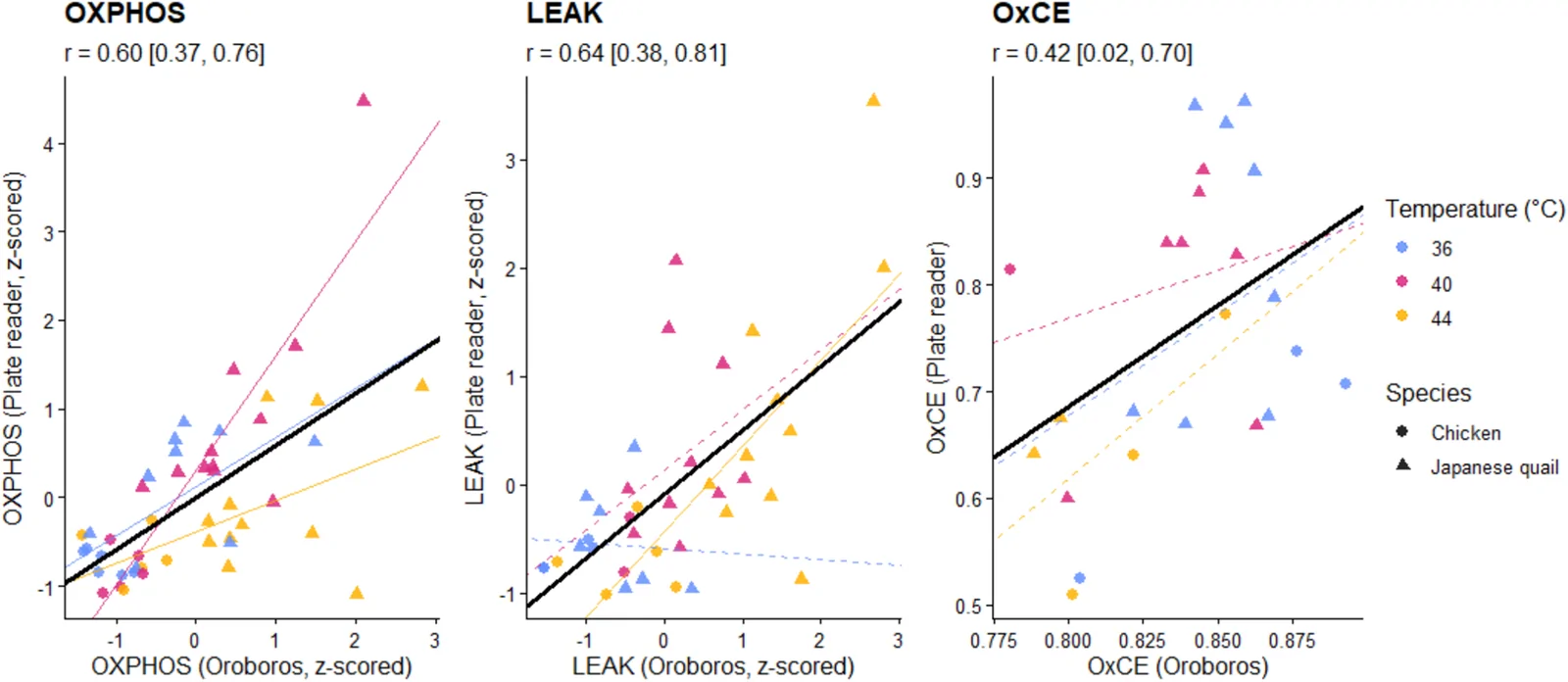
**Relationships between cell-count-corrected mitochondrial OXPHOS_CI+CII_ (*n*=47), *LEAK_CI_*_+II_ (*n*=38) and OxCE (*n*=24) measured by the plate-based assay and HRR method in permeabilised blood cells from chicken and Japanese quail across assay temperatures of 36°C (blue), 40°C (pink) and 44°C (gold).** Each data point represents one biological replicate (one individual bird; *N*=19 for OXPHOS_CI+CII_, *N*=18 for LEAK_CI+CII_, *N*=16 for OxCE), each measured once with both methods at each temperature; plate reader values reflect duplicate technical measurements per individual (see Materials and Methods). Pearson's correlation coefficients are reported with 95% CIs for overall correlation (black), based on two-tailed tests. Pearson's correlation coefficients and detailed sample sizes for temperature-specific correlations are reported in the supplementary materials (Table S4). Symbols denote species (chicken: circles; Japanese quail: triangles). Solid lines show ordinary least-squares regression lines and are displayed only when the association was statistically significant. Dashed lines represent non-significant correlations. *P*-values for each correlation are reported in the supplementary materials (Table S4).

#### Comparing species and temperature effects between methods

Fig. 3 provides a descriptive overview of the mitochondrial trait values obtained with the plate-reader and HRR methods for Japanese quail and chicken blood samples measured at hypothermia (36°C), normothermia (40°C), and hyperthermia (44°C) (raw, cell-count-uncorrected values are shown in Fig. S1). Across all respiratory states and assay temperature, with Japanese quails showing markedly higher OXPHOS_CI+CII_ and LEAK_CI+CII_ than do chickens (Table 1; significant ‘species’ effects were observed for OXPHOS_CI+CII_ and LEAK_CI+CII_ in both methods, but only for OxCE in HRR). In contrast, the effect of temperature differed between methods. Using HRR showed that temperature had significant effects on mitochondrial traits, including the main effect of temperature for LEAK_CI+CII_ (χ^2^=6.59, d.f.=2, *P*=0.037) and OxCE (χ^2^=8.12, d.f.=2, *P*=0.017) and the significant temperature×species interactions for LEAK_CI+CII_ (χ^2^=32.36, d.f.=2, *P*<0.001) and OxCE (χ^2^=7.94, d.f.=2, *P*=0.018). The plate reader detected no significant temperature effects, either as main effects or in interaction with species, for any mitochondrial traits (all *P*>0.10; Table 1).

**Fig. 3.**
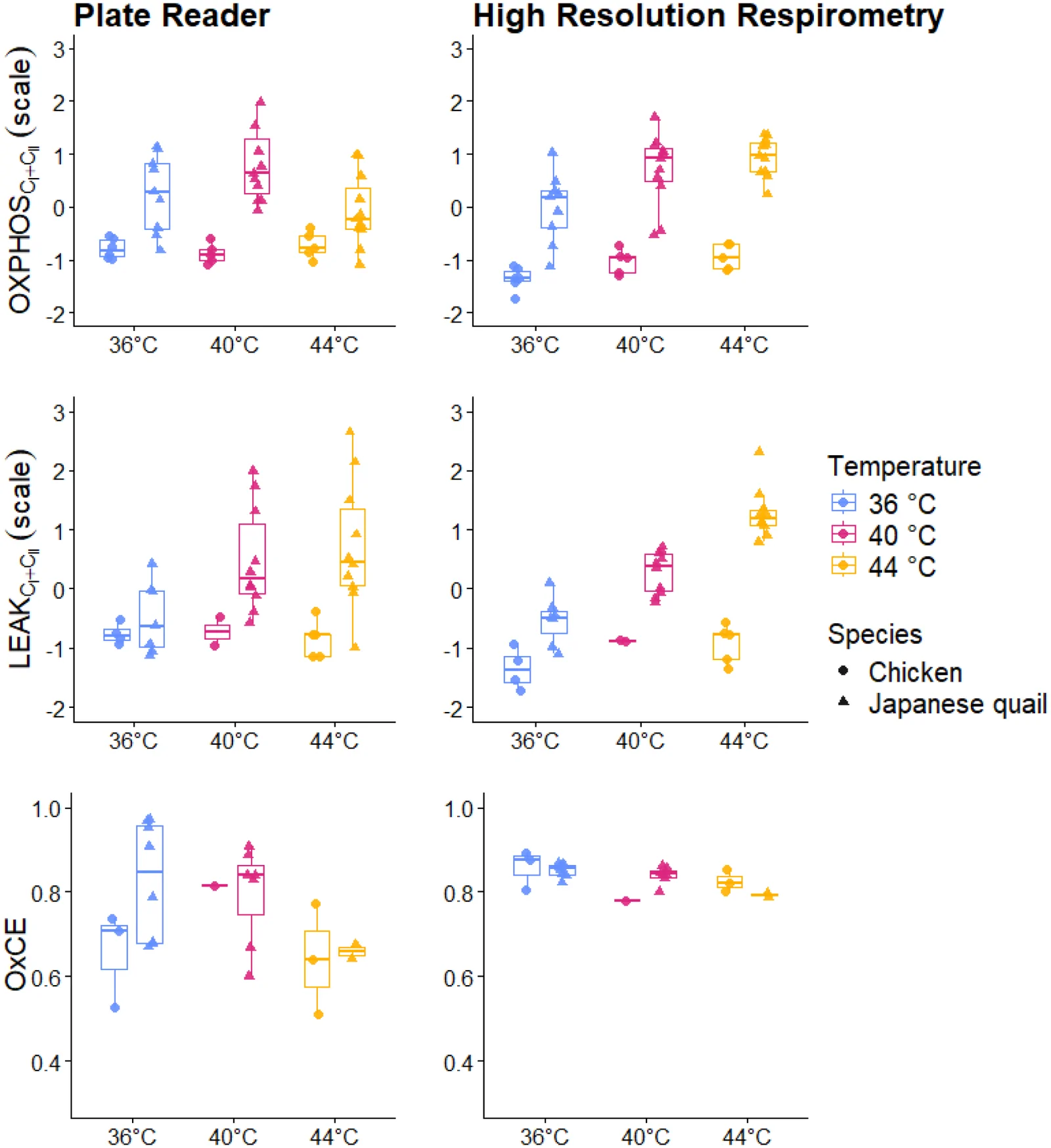
**Data spread depicting assay temperature and mitochondrial respiration rates in avian blood cells measured by fluorescence on a plate reader (left panels) or by HRR on an Oroboros (right panels).** Boxplots show OXPHOS_CI+CII_ (*n*=47, top panels), LEAK*_C_*_I+II_ (*n*=38, middle panels), and OxCE (*n*=24, bottom panels) for chicken (circles) and Japanese quail (triangles) measured at three temperatures (36°C, blue; 40°C, pink; 44°C, gold). Each point represents one biological replicate (one individual bird; *N*=19, 18 and 16 for OXPHOS_CI+CII_, LEAK_CI+CII_ and OxCE, respectively). Points represent individual measurements; boxes indicate interquartile range and median. Respiration values (OXPHOS_CI+CII_, LEAK_CI+II_) were scaled to facilitate cross-method comparison. Statistical results (linear mixed models, two-tailed Type III Wald χ^2^ tests) are reported in Table 2.

**Table 1.** Type III analysis of variance (ANOVA) (Wald χ^2^) tests from linear mixed-effect models assessing the effects of temperature (36°C, 40°C, 44°C), species (chicken, Japanese quail) and their interaction on mitochondrial respiration parameters (OXPHOS_CI+CII_, LEAK_CI+CII_, OxCE) measured with the plate reader and HRR

		Plate reader			HRR		
		χ^2^	d.f.	*P*	χ^2^	d.f.	*P*
**OXPHOS_CI+CII_**	Temperature	0.08	2	0.959	2.65	2	0.266
*N*=19, *n*=47	Species	**8.69**	**1**	**0.003**	**30.05**	**1**	**<0.001**
	Cell count	0.03	1	0.874	1.80	1	0.179
	Temperature:species	3.12	2	0.210	3.91	2	0.142
**LEAK_CI+CII_**	Temperature	0.14	2	0.931	**6.59**	**2**	**0.037**
*N*=18, *n*=38	Species	**5.60**	**1**	**0.018**	**13.55**	**1**	**<0.001**
	Cell count	2.40	1	0.121	0.53	1	0.468
	Temperature:species	4.31	2	0.116	**32.36**	**2**	**<0.001**
**OxCE**	Temperature	1.56	2	0.458	**8.12**	**2**	**0.017**
*N*=16, *n*=24	Species	0.02	1	0.889	**5.48**	**1**	**0.019**
	Temperature:species	2.00	2	0.367	**7.94**	**2**	**0.018**

The analytical models included temperature, species, and their interaction as fixed effects, using cell count as a covariate for both OXPHOS_CI+CII_ and LEAK_CI+CII_ measurements, alongside the method-specific random effects (plate ID or Oroboros-chamber ID plus sample ID). The reported statistics include chi-square values (χ^2^), degrees of freedom (d.f.) and *P*-values for each fixed effect.

Note: χ^2^ statistics are reported from ANOVA type III (two-tailed Wald tests); *P*<0.05 is in bold. *N*=number of individuals, *n*=number of observations.

**Table 2.** Estimates from linear mixed-effect models and Type III ANOVA (Wald χ^2^) tests assessing the effects of sex (male, female) on mitochondrial respiration parameters (OXPHOS_CI+CII_, LEAK_CI+CII_, OxCE) measured with the plate reader and HRR

		Plate reader				HRR			
		Estimates [CI]	χ^2^	d.f.	*P*	Estimates [CI]	χ^2^	d.f.	*P*
**OXPHOS_CI+CII_** *n*=19	Sex	−0.84 [−1.70, 0.01]	3.53	1	0.060	**−0.74 [−1.44, −0.04]**	**4.00**	**1**	**0.046**
	Cell count		0.14	1	0.705		**13.79**	**1**	**<0.001**
**LEAK_CI+CII_** *n*=15	Sex	0.89 [−0.17, 1.95]	2.46	1	0.117	−0.44 [−1.05, 0.18]	1.76	1	0.184
	Cell count		0.39	1	0.527		**30.8**	**1**	**<0.001**
**OxCE** *n*=16	Sex	**−0.11 [−0.19, −0.01]**	**5.45**	**1**	**0.020**	**−0.02 [−0.04, 0.00]**	**4.63**	**1**	**0.030**

The analytical models included sex as a fixed effect, using cell count as a covariate for OXPHOS_CI+CII_ and LEAK_CI+CII_ measurements, alongside the method-specific random effects (plate ID or Oroboros-chamber ID). The reported statistics comprise estimates derived from linear mixed models output alongside their corresponding CIs (reference level: male), chi-square values (χ^2^), degrees of freedom (d.f.) and *P*-values for each fixed effect.

Note: χ^2^ statistics are reported from ANOVA Type III (two-tailed Wald tests); *P*<0.05 is in bold. *n*=number of individuals (one measurement per bird).

Two complementary explanations account for this discrepancy between methods. First, F-tests comparing residual variances from the linear mixed models consistently revealed higher residual variance in plate-reader measurements than in HRR, indicating lower precision as shown by the wider data spread around estimates in Fig. 4. This difference was moderate for OXPHOS_CI+CII_ (F=2.08, *P*=0.014), stronger for LEAK_CI+CII_ (F=11.29, *P*<0.001), and most pronounced for OxCE (F=29.27, *P*<0.001). Second, species-specific models (Fig. 4; Tables S5 and S6) revealed how this reduced precision affected temperature contrasts. Under hypothermia (36°C versus 40°C), both methods generally showed negative estimates for OXPHOS_CI+CII_ and LEAK_CI+CII_ in Japanese quails (Fig. 4C), whereas in chickens, the two methods disagreed not only in magnitude but also in direction: for OXPHOS_CI+CII_, the plate-reader estimate was positive and non-significant, whereas HRR showed a significant negative effect (Fig. 4A). This indicates that reductions in respiratory capacity at lower temperatures were reliably captured by both methods only in quails. Under hyperthermia (44°C versus 40°C), estimates differed in both magnitude and direction between methods and species. Notably, for LEAK_CI+CII_ in Japanese quails under hyperthermia, HRR detected a strong positive effect, whereas the plate-reader estimate remained close to zero with wide uncertainty (Fig. 4D). For OXPHOS_CI+CII_ under hyperthermia, the two methods also diverged: the plate reader indicated a negative effect in Japanese quails, while HRR estimates were close to zero in both species (Fig. 4B,D). For OxCE, no significant effect was observed in chickens under hyperthermia or hypothermia. For Japanese quail OxCE, no significant effects were detected under hypothermia in either method, though estimates varied across them (Fig. 4C). Under hyperthermia, both methods indicated a negative effect, but only the HRR estimate reached statistical significance (Fig. 4B,D). CIs were consistently wider for plate-reader estimates than for HRR estimates across all traits and species, reinforcing the lower sensitivity of this method to detect temperature effects.

**Fig. 4.**
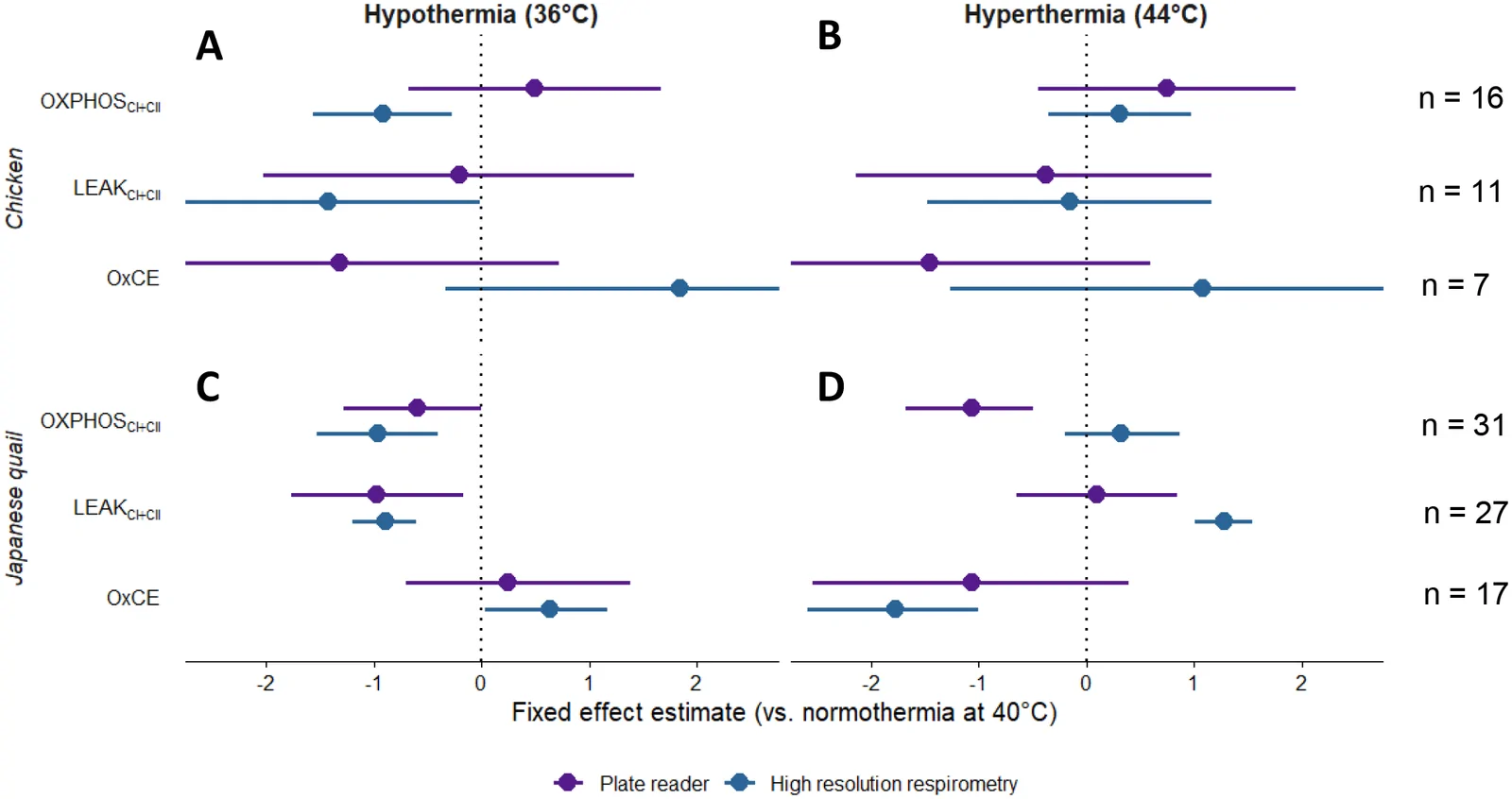
**Fixed-effect estimates (±95% CI) from linear mixed models comparing mitochondrial traits (OXPHOS_CI+CII_, LEAKC_I+CII_, OxCE) between hypothermia (36°C) or hyperthermia (44°C) and normothermia (40°C) in chicken (A,B) and Japanese quail (C,D), measured by plate-reader and HRR.** Each point represents one biological replicate (one individual bird). Chicken: *N*=6 (OXPHOS_CI+CII_), *N*=5 (LEAK_CI+CII_), *N*=7 (OxCE); Japanese quail: *N*=13 (OXPHOS_CI+CII_), *N*=13 (LEAK_CI+CII_), *N*=12 (OxCE). Model summaries are provided in the supplementary material as Table S5 for chicken and Table S6 for Japanese quail. Test results (linear mixed models, two-tailed *t*-tests on fixed effects) are reported in Tables S5 and S6. Mitochondrial traits were scaled before analyses to allow comparison of effect sizes between traits and species. OxCE estimates are multiplied by 10 for visual comparability with the other traits.

An analysis using the full sample size for both instruments was conducted, with results similar to those presented above (see Tables S7 and S8). Because sample sizes were smaller for chickens than for Japanese quails, the estimates for chickens were less precise (i.e. associated with larger CIs in Fig. 4).

### Experiment 2: Within-species variation

#### Correlations between the plate-reader and HRR methods

Measurements of mitochondrial traits obtained with the plate-based method were positively correlated with those obtained from the HRR method in quails (Fig. 5). Pearson's correlation coefficients were 0.52 (95% CI [0.10, 0.79]; *n*=19) for OXPHOS_CI+CII_, 0.12 (95% CI [−0.40, 0.60]; *n*=15) for LEAK_CI+CII_, and 0.44 (95% CI [0.01, 0.77]; *n*=16) for OxCE. Correlations were statistically significant for OXPHOS_CI+CII_ and OxCE, but not for LEAK_CI+CII_.

**Fig. 5.**
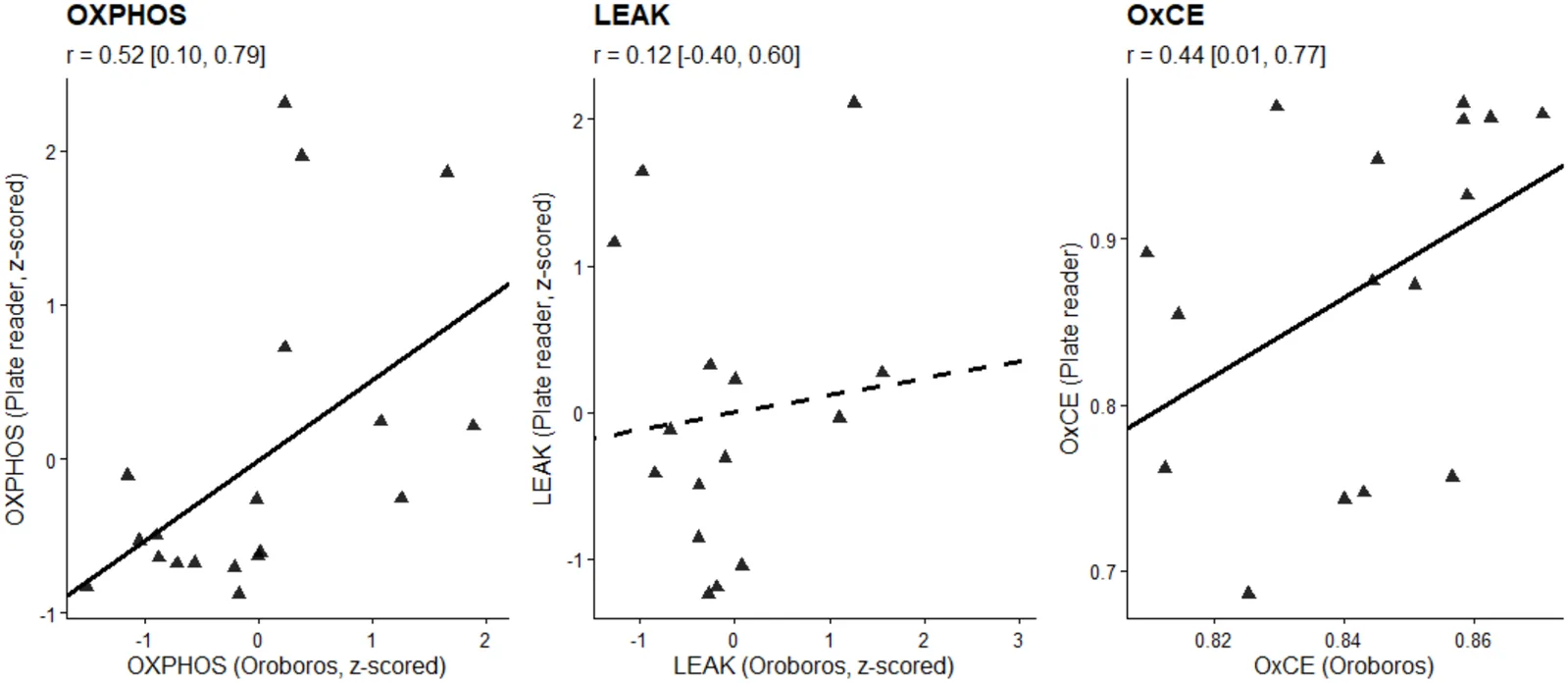
**Relationships between cell-count-corrected mitochondrial OXPHOS_CI+CII_ (*n*=19), LEAK_CI+II_ (*n*=15) and OxCE (*n*=16) measured by the plate-based assay and HRR methods in permeabilised blood cells from Japanese quail in a single experiment.** Each data point represents one biological replicate (one individual quail), each measured once with both methods; plate-reader values reflect duplicate technical measurements per individual (see Materials and Methods). Pearson's correlation coefficients are reported with 95% CIs, based on two-tailed tests. Exact *P*-values are reported in the main text (see Results). Solid lines show ordinary least-squares regression lines and are displayed only when the association was statistically significant. Dashed lines represent non-significant correlations.

#### Biological comparison of the plate-reader and HRR measurements

Across all respiratory states, the two methods provided consistent results when examining the effect of sex on LEAK_CI+CII_ and OxCE and showed a similar pattern for OXPHOS_CI+CII_, though only HRR reached statistical significance (Table 2, Fig. 6; raw values in Fig. S2). For OXPHOS_CI+CII_, both methods yielded similar effect sizes (plate reader: estimate=−0.84 [−1.70, 0.01], χ^2^=3.53, d.f.=1, *P*=0.060; HRR: estimate=−0.74 [−1.44, −0.04], χ^2^=4.00, d.f.=1, *P*=0.046), with HRR estimates narrowly reaching statistical significance and the plate reader estimates falling just short, in the same direction. For LEAK_CI+CII_, sex was not significant with either method (plate reader: χ^2^=2.46, d.f.=1, *P*=0.117; HRR: χ^2^=1.76, d.f.=1, *P*=0.184). For OxCE, sex was significant with both methods (plate reader: χ^2^=5.45, *P*=0.020; HRR: χ^2^=4.63, d.f.=1, *P*=0.030).

**Fig. 6.**
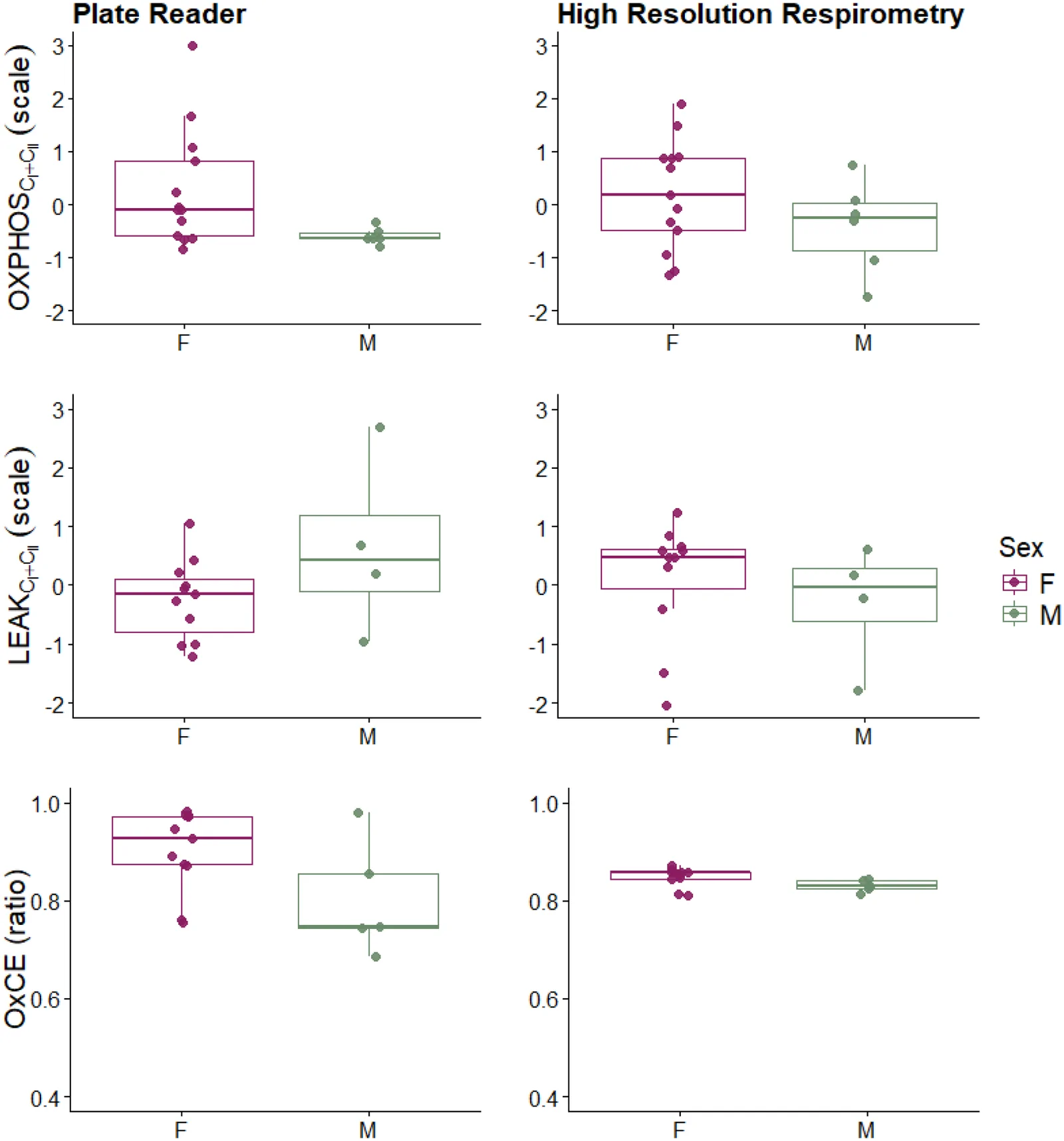
**Data spread depicting sex differences and mitochondrial respiration rates in avian blood cells measured by fluorescence on a plate reader (left panels) or by HRR on an Oroboros (right panels).** Boxplots show OXPHOS_CI+CII_ (*n*=19, top panels), LEAK*_C_*_I+II_ (*n*=15, middle panels), and OxCE (*n*=16, bottom panels) measured at 40°C. Each point represents one biological replicate (one individual quail). Points represent individual measurements; boxes indicate interquartile range and median. Respiration values (OXPHOS_CI+CII_, LEAK*_C_*_I+II_) were scaled to facilitate cross-method comparison. Statistical results (linear mixed models, two-tailed Type III Wald χ^2^ tests) are reported in Table 2.

### Pooled analysis across experiments: correlation between the plate-reader and HRR methods at normothermia

When combining measurements of mitochondrial traits at normothermia from Experiments 1 and 2, results obtained with the plate-based method were positively correlated with those obtained using the HRR method (Fig. 7). Pearson's correlation coefficients were 0.49 (95% CI [0.13, 0.74]; *n*=26) for OXPHOS*_C_*_I+II_, 0.22 (95% CI [−0.19, 0.57]; *n*=24) for LEAK_CI+II_, and 0.43 (95% CI [0.03, 0.72]; *n*=24) for OxCE. Correlations were statistically significant for OXPHOS*_C_*_I+II_ and OxCE, but not for LEAK_CI+II_.

**Fig. 7.**
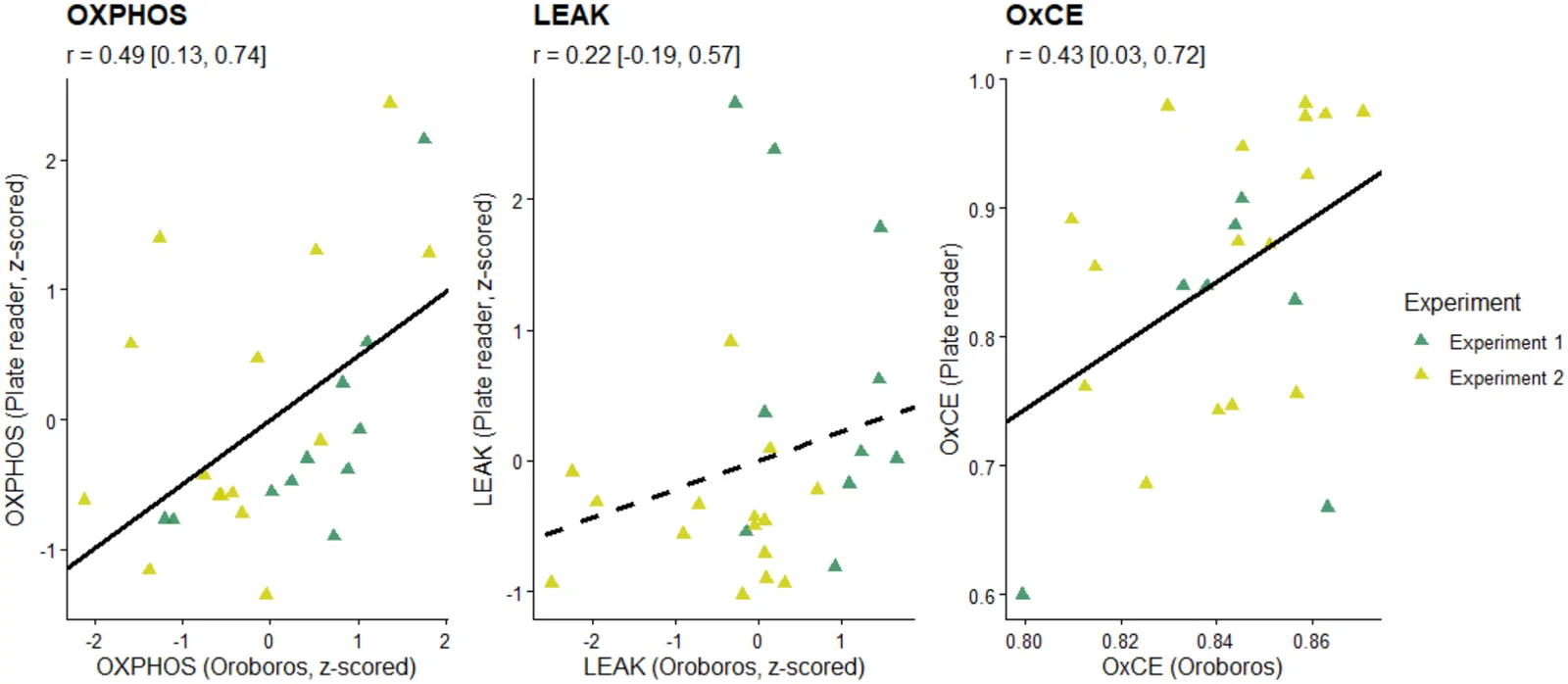
**Relationships between cell-count-corrected mitochondrial OXPHOS_CI+CII_ (*n*=26), LEAK_CI+II_ (*n*=24) and OxCE (*n*=24) measured by the plate-based assay and HRR methods in permeabilised blood cells from Japanese quail combining Experiments 1 (green) and 2 (yellow) at normothermia (40°C).** Each data point represents one biological replicate (one individual quail; *N*=*n*, as each bird contributed a single measurement at 40°C, with no individuals shared between Experiments 1 and 2). Pearson's correlation coefficients are reported with 95% CIs, based on two-tailed tests. Solid lines show ordinary least-squares regression lines and are displayed only when the association was statistically significant. Dashed lines represent non-significant correlations.

### Quality check of mitochondrial respiration during plate-based measurements

Consistent with the results of Experiment 1, further assessment of mitochondrial integrity using the proposed plate-reader protocol showed a significant difference across assay temperatures in the proportion of individual×temperature comparisons exceeding the 10% membrane-integrity threshold (χ^2^=6.04, d.f.=2, *P*=0.049). The average increase in OXPHOS_CI+CII_ following the addition of cytochrome c increased with assay temperature (mean±s.e.: 3.03±1.52% at 36°C, 5.83±1.93% at 40°C, and 7.64±2.01% at 44°C). There was no evidence of a meaningful (>10%) increase in OXPHOS_CI+CII_ following the addition of cytochrome c at 40°C (zero of 12 measurements), and one of 12 (8.3%) measurements exceeded this threshold at 36°C, compared with four of 12 (33.3%) at 44°C (Fig. S4).

## DISCUSSION

Overall, agreement between the plate-based assay and HRR was condition dependent, with performance varying by assay temperature and respiratory state rather than showing broadly similar patterns across the board. In particular, we found, in Experiment 1, that plate-reader measurements showed high technical repeatability at 36°C and 40°C and produced significantly positive correlations with HRR estimates for OXPHOS_CI+CII_ and LEAK_CI+II_ (Fig. 2), and both methods detected interspecific differences at the three temperatures (Table 1). These results suggest that, around normothermic conditions (40°C), the plate reader can recover comparable patterns (e.g. species differences) for OXPHOS and LEAK respiration rates, which supports its use as a valid method when high-throughput measurements are required and a large effect size is expected.

However, the correlations were somewhat weaker for OxCE, and discrepancies between methods emerged mainly while mimicking hypothermia (36°C) under low O_2_ flux (i.e. chickens) and under hyperthermic (44°C) assay temperatures, where the plate reader showed larger residual variance and wider CIs than did HRR (Table 1; Fig. 4). Overall significant temperature effects were detected only with HRR, except for Japanese quails at 36°C versus 40°C. Complementary analyses assessing mitochondrial integrity under the plate-reader protocol by adding cytochrome c to OXPHOS respiration showed a higher proportion of samples exceeding the integrity threshold at 44°C than at 36°C or 40°C (see Results section), offering a plausible additional explanation for the reduced agreement between methods at 44°C, alongside the lower sensitivity of the plate reader noted above. Taken together, these results are broadly consistent, with the plate-reader approach performing most reliably under stable, normothermic conditions.

Accordingly, when comparing different types of biological variation under normothermic conditions in Experiment 2, the two methods reached consistent conclusions regarding the effects of sex on LEAK (no effect detected by either method) and OxCE (significant with both methods) and showed comparable effect sizes for OXPHOS, although this effect narrowly reached significance only with HRR. Thus, these two techniques are not interchangeable in all contexts, and the method choice should be guided by the biological question, the respiratory traits of interest, and the required sensitivity to detect temperature effects or subtle variations.

### Comparison of the plate-reader approach to the gold-standard HRR approach

The comparison between this plate-based method and the gold-standard HRR method suggests that the two approaches are not fully interchangeable. HRR remains the preferred option when high sensitivity, fine temporal resolution, and detailed characterisation of mitochondrial aerobic metabolism at low fluxes are required, for example, to detect subtle among-individual differences, small treatment effects, or responses at non-optimal temperatures (Awadhpersad and Jackson, 2021; Coulson et al., 2024; Djafarzadeh and Jakob, 2017b). In contrast, the plate reader prioritises throughput over very high resolution, as multiple wells can be processed simultaneously, enabling parallel data acquisition from numerous individuals within the same physiological state. This is expected to enhance technical repeatability, as samples are exposed to identical environmental conditions and substrate additions within a single plate. While the observed high repeatability of OXPHOS_CI+CII_ and LEAK_CI+II_ respirations in this study indicates that wells behaved consistently under identical conditions, this alone does not confirm robustness but rather provides a necessary foundation to assess it. This robustness could nonetheless be compromised by edge effects, whereby wells positioned at the periphery of a plate can experience thermal and evaporative conditions different from those experienced by interior wells (Lundholt et al., 2003); appropriate positional controls are therefore needed to confirm that duplicate measurements remain comparable across a plate, a factor not formally assessed in the present study. By contrast, OxCE showed much lower and more uncertain repeatability, underscoring that not all derived mitochondrial traits are equally well suited for this plate-based implementation. Consistent patterns observed in other avian systems, such as white-throated dippers (*Cinclus cinclus*; M. Ohse, unpublished) and alpine swifts (*Tachymarptis melba*; C. Hadjadji, unpublished), further support the potential robustness of this high-throughput approach.

From a practical perspective, the plate reader's advantages are best understood as a combination of cost and throughput rather than either alone. Polarographic (Clark-type) electrodes remain the least expensive option for measuring mitochondrial oxygen consumption, but, like HRR, they measure only one sample at a time, and their comparatively low sensitivity typically requires a higher mitochondrial density or respiratory flux to obtain a reliable signal (Silva and Oliveira, 2012). HRR itself, while more precise, carries a high initial cost and remains similarly low throughput; and dedicated high-throughput systems such as the Seahorse XF analyser are both specialised and expensive. The plate reader is, to the best of our knowledge, the only current approach combining relatively low cost with high throughput. While the initial investment in the instrument and fluorescent oxygen probe is high, the per-sample running costs remain minimal (Divakaruni and Jastroch, 2022). The same plate reader can also be used for a wide range of fluorescence- or absorbance-based biochemical assays, increasing its overall utility and availability in typical laboratory environments, beyond this specific application. The ability to process 13–15 samples simultaneously can represent up to a sixfold or sevenfold increase in efficiency compared to standard high-resolution chambers, which typically allow two measurements at a time unless multiple instruments are available (Djafarzadeh and Jakob, 2017a). While the initial cost of the instrument is high, there is only one plate reader needed to sustain this high-throughput method. Finally, in contrast to HRR methods, the plate reader does not require continuous supervision and can operate autonomously once the plate has been prepared and loaded, which may be advantageous in settings with limited specialised personnel or time.

The two approaches were also comparable in their ability to capture some biologically relevant variations, especially for interspecific differences in LEAK and OXPHOS at each temperature. Both methods led to the same conclusions regarding interspecific differences in mitochondrial respiration rates (Table 2, Fig. 3) and, for OXPHOS_CI+CII_ and LEAK_CI+II_, tended to show similar contrasts between 36°C and 40°C in Japanese quails (Fig. 4), indicating that the plate reader can yield comparable qualitative biological inferences when measurements are taken at these temperatures and respiratory fluxes are relatively high. Similarly, in Experiment 2, the two methods reached consistent conclusions regarding the effects of sex for LEAK_CI+CII_ and OxCE in quails under normothermic conditions. For OXPHOS_CI+CII_, the two methods yielded similar effect sizes (plate reader: β=−0.84; HRR: β=−0.74), but the effect size reached significance only with HRR (*P*=0.046), while the plate reader showed a comparable trend that fell just short of significance (*P*=0.060). This pattern is consistent with the plate reader's generally lower sensitivity due to greater measurement error (Divakaruni and Jastroch, 2022), rather than a true biological discrepancy between methods, and illustrates how comparable underlying effects can fall on either side of a significance threshold depending on assay precision. Thus, the finer resolution of HRR instruments allows for more sensitive detection of variation among individuals, particularly for effects close to the detection limit of the plate-based assay, and this limitation was especially apparent in our chicken data from Experiment 1, where lower respiration rates resulted in larger residual variance and wider CIs for plate-reader estimates (Table 1, Fig. 4).

At higher temperatures (e.g. 44°C), the cells are exposed to the pre-warmed mineral oil and plate-reader chamber for approximately 30 s to 1 min before the first recorded reading, which may initiate mitochondrial stress that continues to compound over the subsequent 90-min measurement window as cells remain at this elevated temperature throughout the run. HRR measurements, by contrast, are taken immediately after substrate addition within a continuously monitored, temperature-controlled chamber, which may better preserve cellular integrity under these more thermally demanding conditions (Stier et al., 2017; Djafarzadeh and Jakob, 2017a). This interpretation is consistent with the mitochondrial integrity findings reported above (Fig. S4), where compromise of the outer mitochondrial membrane was more frequent at 44°C than at lower temperatures. Together, these patterns suggest that HRR is better suited for exploring mitochondrial performance at the edges of the thermal or metabolic envelope, whereas the plate reader may be reliable for characterising variation under near-physiological temperatures and sufficiently high-flux conditions.

The comparison of flux control ratios such as OxCE between both methods did not lead to straightforward, similar biological interpretations (Fig. 3). We had anticipated this outcome to some extent due to the difference in resolution between the plate reader and HRR and because OxCE has intrinsically lower technical repeatability than respiration rates as it accumulates measurement error in both terms of the ratio, even with HRR methods (Nord et al., 2023). In Experiment 1, plate-based measurements tended to yield systematically lower OxCE values than did HRR (Fig. 3). Because OxCE is a ratio, this cannot simply reflect the plate reader's generally lower OXPHOS and LEAK values overall but rather points to a disproportionate difference in accuracy between the two traits. Notably, this underestimation was not apparent in Experiment 2, where plate-reader OxCE values were more closely aligned with those from HRR (Fig. 6). This discrepancy between experiments suggests that the divergence in OxCE observed in Experiment 1 may be linked to the wider range of assay temperatures tested, rather than reflecting an inherent bias of the plate-reader approach under normothermic conditions. Plate-reader OXPHOS and OxCE both correlated significantly with HRR across Experiment 1, Experiment 2, and the pooled 40°C dataset (Figs 2, 5, and 7), whereas LEAK correlated significantly only in Experiment 1. This systematic underestimation of OxCE on the plate reader, despite OXPHOS tracking HRR closely, is consistent with LEAK being relatively overestimated by this method, rather than reflecting a general loss of resolution in the underlying respiration measurements. In other species, for which parallel HRR measurements are not available, we have observed that the technical repeatability of OxCE across duplicate plate-reader wells improves with higher respiration rates (C. Hadjadji and M. Ohse, unpublished), further suggesting that its reduced concordance with HRR at low fluxes reflects measurement sensitivity rather than a fundamental incompatibility between platforms. Despite this, methodological reviews on dedicated plate-based systems such as Seahorse XF analysers suggest ratio-based parameters [e.g. respiratory control ratio (RCR) and coupling efficiency] are considered most useful for detecting differences among treatments or model systems within a given platform and require some caution when compared quantitatively with values obtained using chamber-based instruments (Divakaruni and Jastroch, 2022; Gerencser et al., 2009; Zdrazilova et al., 2022).

### Improvements to the plate-reader method

Future refinements could further improve the comparability and analytical power of plate-based respirometry. Developing a true baseline or ‘zero’ oxygen calibration would permit conversion of fluorescence units into absolute oxygen consumption rates (Gnaiger, 2021, http://wiki.oroboros.at/index.php/MiPNet06.03_POS-Calibration-SOP; Gerencser et al., 2009; Divakaruni and Jastroch, 2022). This step would standardise plate-reader outputs with established HRR units, facilitating direct cross-study comparisons. Additionally, replacing the mineral oil sealing system with a controlled atmospheric chamber could enable the reuse of wells for downstream assays, such as quantifying ATP concentration or reactive oxygen species production from the same sample (Yu et al., 2021; Divakaruni and Jastroch, 2022). Current limitations primarily relate to sensitivity. The extracellular oxygen probe used in this study (e.g. MitoXpress™ Xtra, Agilent Technologies, Santa Clara, CA, USA) detects larger changes in oxygen concentration rather than direct intracellular respiration rates (Agilent Technologies, 2019, https://www.agilent.com/cs/library/usermanuals/public/5991-9328EN_MitoXpress_Xtra_User_Guide.pdf). Consequently, states such as endogenous cellular respiration (i.e. *ROUTINE*) remain undetectable at present. The use of intracellular probes, such as MitoXpress™ Intra (Agilent Technologies, Santa Clara, CA, USA), could help address this limitation and may expand the range of measurable mitochondrial parameters without substantially altering the assay design (Agilent Technologies, 2019, https://www.agilent.com/cs/library/usermanuals/public/5991-9327EN_MitoXpress_Intra_User_Guide.pdf). The 0.50 OxCE threshold applied here was intended to exclude only clearly non-functional preparations, not to serve as a finely tuned diagnostic cut-off; validating a more precise threshold against an independent measure of mitochondrial function would be a valuable next step. Such developments would further clarify the range of conditions under which plate-based measurements can robustly complement HRR, particularly for traits that currently sit near the detection limit of the assay. Further protocol optimisation for each study system could focus on several aspects of the assay's data extraction and preparation to improve measures from this plate-reader approach, especially for lower-flux traits such as LEAK, which is slower and of smaller magnitude than OXPHOS. Increasing the raw signal magnitude, for example, by loading a greater number of cells per well, could further improve resolution at low respiratory fluxes. Considering our results, improvements that enhance sensitivity at low fluxes and reduce residual variance would be especially valuable.

### Perspectives in ecology and evolution

From an ecological and evolutionary perspective, the high-throughput plate-based approach, if optimised properly, may open up important new opportunities for large-scale and comparative studies of mitochondrial function. The method's scalability and relatively simple sample preparation enable researchers to quantify mitochondrial traits across hundreds of individuals or multiple species within a feasible time frame, a task previously impractical with traditional HRR systems. In addition to interspecific comparisons, our finding that the plate reader could also capture sex-related variation within a single species (Experiment 2) suggests its utility may extend to finer-scale, within-population comparisons relevant to quantitative genetics and life-history research, at least for traits where the effect sizes were comparable between methods even when formal significance depended on assay precision (OXPHOS).

At the same time, we do not expect a single, universal relationship between plate-reader and HRR measurements across all species and conditions. Variation in mitochondrial respiration intensity (e.g. between species in this study or between life stages; Cossin-Sevrin et al., 2022), blood cell composition, mitochondrial volume/density, mitochondrial organisation, coupling efficiency, and species-specific thermal physiology could all influence how closely the two approaches mirror one another. As this protocol continues to be developed and extended to additional species, taxa, and conditions, a broader comparison against HRR would be a valuable next step to better characterise how consistently the two approaches align and to identify the biological or methodological factors that shape this correspondence. This advancement is especially relevant for data-intensive studies exploring, for example, the heritability of mitochondrial performance, population-level physiological diversity, or the relationship between metabolism and fitness in wild or semi-controlled conditions (Rønning et al., 2007; Stier et al., 2019; Casagrande and Dell'Omo, 2025; Correia et al., 2025; Salin et al., 2019).

By facilitating simultaneous measurement of diverse metabolic substrates, this approach may also provide a promising avenue for integrative research on substrate utilisation and physiological flexibility. Given the assay's reduced sensitivity to temperature effects reported here, we suggest that future applications avoid temperature tolerance assays, which showed limited performance at high temperatures with the current implementation, and instead focus on comparative or dose-response studies that do not rely on extreme thermal conditions. For instance, lipid- versus carbohydrate-based substrate protocols could be tested on the same plate, allowing researchers to explore metabolic trade-offs and substrate use with ecological or evolutionary relevance. Moreover, the plate format enables toxicity screening and dose-dependent assays, providing a high-throughput platform for studying the effects of environmental contaminants or metabolic inhibitors. An advantage of HRR is the very low reaction volume, which minimises the amount of biological material required. This feature is particularly beneficial when sampling small species or rare tissues, and it makes the method easily adaptable to other cell types or isolated mitochondria beyond avian blood cells. With further optimisation, the plate-reader approach could achieve comparable performance under a wider range of conditions. Such flexibility could broaden its use in comparative mitochondrial studies across taxa, helping to illuminate mechanisms of energy allocation, physiological adaptation, and metabolic diversity. More broadly, after appropriate optimisation, the plate-reader approach described here should be applicable to other tissues and species with sufficiently high O_2_ fluxes.

In summary, the present study suggests that a plate-based assay could be used alongside HRR to measure mitochondrial aerobic metabolism under normothermic conditions, but not as a replacement for HRR. Its high technical repeatability of OXPHOS and LEAK and positive cross-method correlations of OXPHOS and OxCE across both experiments and the pooled dataset are encouraging for its use as a complementary tool. It also captured interspecific differences (Experiment 1) and intraspecific sex-related differences for OxCE, along with comparable OXPHOS effect sizes (Experiment 2). Together with its low operational cost and scalability, these strengths could facilitate the expansion of mitochondrial research into ecological and evolutionary frameworks under near-normothermic conditions. However, its lower precision compared to HRR, reduced sensitivity to temperature effects and to low-flux traits such as LEAK, as well as OxCE's lower technical repeatability and the method's divergent detection of temperature effects, mean its use should, for now, be restricted to the conditions validated here: near-normothermic temperatures and sufficiently high respiration rates. Further validation is needed under other experimental conditions.

## MATERIALS AND METHODS

The species husbandry conditions, blood sampling procedures, blood processing methods, cell count quantification, reagent preparation, and data cleaning criteria were identical between Experiments 1 and 2, with the exception of species: Experiment 1 included both Japanese quails (*Coturnix japonica*; Temminck & Schlegel, 1849) and chickens (*Gallus gallus domesticus*; Linnaeus, 1758), whereas Experiment 2 only used quails (see Fig. 1 for a graphical summary of both experiments).

### Model species

Japanese quails (*C. japonica*; Temminck & Schlegel, 1849), hereafter quails, and chickens (*G. gallus domesticus*; Linnaeus, 1758) are two of the most commonly used species in avian and poultry research due to their economic importance and ease of rearing. Indeed, their rapid maturation, small body size, and low maintenance requirements make them ideal for laboratory studies. For Experiment 1, we obtained whole blood from 13 adult quails and six adult chickens. For Experiment 2, we obtained whole blood from 19 adult quails, comprising 13 females and six males. The quails were sourced from INRAE Nouzilly in France and were housed at IPHC-Strasbourg for at least 20 weeks prior to sampling, in a large outdoor aviary measuring 8×16×3 m. Blood samples (∼300 μl) were drawn from the wing vein of the quails using heparinised capillaries and stored on ice until further processing (within a maximum of 2 h); this procedure was carried out under the ethical permit APAFIS #54114-2025030516541037 v4. Blood samples from the chickens were obtained from a local slaughterhouse in heparinised tubes and then immediately placed on ice until further processing (a maximum of 3 h).

### Blood sample preparation

On each experimental day, blood was collected fresh from a different subset of individuals in the morning and measured sequentially throughout the day to ensure that it was never stored overnight or reused across days. For Experiment 1, we added 240 μl of whole blood per individual into 4.26 ml of MiR05 buffer [0.5 mM ethylene glycol-bis(β-aminoethyl ether)-N,N,N′,N′-tetraacetic acid (EGTA), 3 mM MgCl_2_, 60 mM K-lactobionate, 20 mM taurine, 10 mM KH_2_PO_4_, 20 mM HEPES, 110 mM sucrose, free fatty acid BSA (1 g l^−1^), pH 7.1; Sigma-Aldrich, St. Louis, MO, USA] following the protocol of Fasching et al. (2016) and Oroboros Instruments (Innsbruck, Austria). This stock sample solution was then divided into 3×0.8 ml for the HRR method (i.e. 42.7 μl of blood per assay in a chamber of 2 ml), 1.24 ml for the plate-reader method, and 20 μl for cell count analyses. For Experiment 2, we added 100 µl of blood per individual into 3 ml of MiR05 buffer, where 2 ml was used for HRR measurements, 300 µl was used for the plate reader, and 20 µl was used for cell count analyses.

### Mitochondrial measurements

#### Equipment, assay parameters and reagents

For the plate-reader method, we used the VANTAstar microplate reader from BMG Labtech GmbH (Ortenberg, Germany). Mitochondrial oxygen consumption was measured using the MitoXpress™ Xtra Oxygen Consumption Assay (Agilent Technologies, Santa Clara, CA, USA). The plate reader was configured for MitoXpress™ Xtra measurements in fluorescence mode, using an excitation filter (Ex TR), a dichroic filter (LP TR) and an emission filter (645/20 nm). For the HRR method, we used three O2k HRRs from Oroboros Instruments (Innsbruck, Austria).

Both the VANTAstar microplate reader and the HRR Oroboros have temperature-controlled chambers which were set to the required temperature prior to the start of measurement. For Experiment 1, blood samples were measured at three different temperatures using both methods: (1) 36°C to mimic hypothermia, (2) 40°C being within normothermic range, and (3) 44°C to mimic hyperthermia. The experiment was conducted over 3 days, with a different subset of individuals being measured each day. On each day, all three temperature runs were performed sequentially using blood collected that morning, ensuring that the full set of measurements for each individual was completed within a single day. To avoid a time-of-day sampling bias, the order of the measurements at each temperature was randomised each day (i.e. on the first day, the order of the measurements for both instruments was 40°C, 44°C and then 36°C, while on the second day, the measurement order for both instruments was 36°C, 40°C, and then 44°C). For Experiment 2, blood samples were measured over 3 days at 40°C (within normothermic range) for both methods.

The reagents used for both the plate-reader and HRR methods are listed in Table 3 and further detailed in Tables S1–S3 (Sigma-Aldrich, St. Louis, MO, USA). This table includes the final titration used in each chamber of the HRR method and in each well of the plate-reader method. The reagents were prepared following SUIT protocols available through Oroboros Instruments (Innsbruck, Austria).

**Table 3.** Titration table adapted for the HRR and plate-reader methods following SUIT protocols from Oroboros Instruments (Innsbruck, Austria)

				HRR			Plate reader		
Substrate	Stock	Solvent	Storage	Chamber	Titration	Final conc.	Well	Titration	Final conc.
	mM		°C	μl	μl	mM	μl	μl	mM
Pyruvate	2000	H_2_O	fresh	2000	5	5	100	0.25	5
Malate	800	H_2_O	−20	2000	5	2	100	0.25	2
Succinate	1000	H_2_O	−20	2000	20	10	100	1	10
ADP	500	H_2_O	−80	2000	5	1.25	100	0.25	1.25
Inhibitor	Stock	Solvent	Storage	Chamber	Titration	Final conc.	Well	Titration	Final conc.
	mM		°C	μl	μl	mM	μl	μl	mM
Oligomycin	5	EtOH	−20	2000	1	2.5	100	0.05	2.5
Antimycin A	5	EtOH	−20	2000	1	2.5	100	0.05	2.5

The final concentrations represent what is present in each chamber of the Oroboros and in each well of the plate-reader assay.

#### Mitochondrial respiration rates and flux ratio

For both methods, we used reagents to measure two respiration rates in blood cells permeabilised with digitonin (10 mg ml^−1^), namely, (i) the maximum respiration rate fuelled with exogenous substrates of Complex I [pyruvate (5 mmol l^−1^), malate (2 mmol l^−1^)] and Complex II [succinate (5 mmol l^−1^)], as well as ADP (1.25 mmol l^−1^; OXPHOS_CI+CII_), and (ii) the respiration rate related to LEAK in the presence of exogenous substrates of Complexes I and II, ADP and oligomycin (2.5 mmol l^−1^; LEAK_CI+II_). Non-mitochondrial respiration was measured in the presence of exogenous substrates of complexes I and II, ADP, and antimycin A (5 mmol l^−1^), and this non-mitochondrial respiration was subtracted from OXPHOS_CI+CII_ and LEAK_CI+II_ respiration rates. Antimycin A was chosen as the inhibitor for this step because, as a specific inhibitor of Complex III, downstream of both Complex I and II, it fully suppresses electron flow from both substrate entry points without affecting non-mitochondrial oxygen-consuming enzymes. In contrast, complex IV inhibitors such as KCN or sodium azide also inhibit haem-containing non-mitochondrial enzymes (e.g. NADPH oxidases and cytochrome P450s), which would confound quantification of residual oxygen consumption (Brand and Nicholls, 2011; Pesta and Gnaiger, 2012). The use of antimycin A for this purpose is consistent with established HRR protocols, including those for avian blood cells (Stier et al., 2017; Nord et al., 2021). All reagent concentrations mentioned in the above text are the same in the following parts of the methods.

To gain insight into the efficiency of mitochondria to produce ATP, we analysed (iii) the OxCE computed as (OXPHOS_CI+CII_−LEAK_CI+CII_)/OXPHOS_CI+CII_ (Gnaiger et al., 2019). OxCE is included as a biologically meaningful relative index capturing the proportion of oxygen consumption devoted to ATP synthesis versus LEAK, interpretable within each platform on its own terms.

#### HRR using the Oroboros: general protocol

HRR was carried out using whole-blood samples with Oroboros O2k instruments (Oroboros Instruments, Innsbruck, Austria), following the approach described by Stier et al. (2019) for avian blood cells. In brief, blood cells that were suspended in MiR05 underwent washing through centrifugation and supernatant removal prior to being loaded into the HRR chambers, which contained MiR05 that had been pre-equilibrated at temperatures of 36°C, 40°C or 44°C, for the purpose of measuring changes in oxygen concentration within the closed chamber. Following a brief equilibration period, the blood cells were permeabilised with 5 μl of digitonin (Sigma-Aldrich, St. Louis, MO, USA). Then, we sequentially added pyruvate/malate and succinate together with ADP to drive OXPHOS_CI+CII_, followed by oligomycin to inhibit ATP synthase and thus isolate LEAK_CI+CII_ and, finally, antimycin A to inhibit Complex III and quantify non-mitochondrial respiration (see Table 1 for titration details). The three Oroboros instruments were calibrated daily for 100% and 0% and before the experiment for background O_2_ flux at each temperature using a sequential addition of dithionite. Each Oroboros was used at the three assay temperatures to avoid confounding effects of Oroboros identity and assay temperature.

#### Microplate-based fluorescent assay using a plate reader: general protocol

Following the preparation of the blood sample detailed above, 300 μl aliquots of blood cells were separated for same-day measurements at the assay temperature(s) relevant to each experiment (Experiment 1: 36°C, 40°C, or 44°C; Experiment 2: 40°C), and 5 μl of digitonin (Sigma-Aldrich, St. Louis, MO, USA) was added to permeabilise the cells. Preparation of respiration buffers involved creating a master mix for all buffers with the overlapping reagents. Required volumes of reagents were calculated based on the number of samples being measured (see supplementary materials). For the assay, 60 μl of blood cell suspension was added to each well, followed by 40 μl of the respective respiration buffer. The final well volume was 100 μl, consisting of 60 μl of permeabilised blood cells in MiR05 and 40 μl of desired respiration buffer. All respiration buffers were loaded in duplicate on the plate, except for the non-mitochondrial respiration buffer, which was loaded in simplicate to maximise the number of samples that could be run on the same plate. Preliminary tests showed that non-mitochondrial respiration exhibited high technical repeatability (>0.95), similar to the other respirations (see below). Finally, the microplate wells were sealed with 100 μl of pre-warmed mineral oil, at 36°C, 40°C, or 44°C, depending on the experiment, to prevent oxygen exchange. We pipetted the mineral oil using 10 ml Eppendorf ViscoTip syringe tips (Hamburg, Germany) to prevent air bubbles forming in the oil layer, as these can affect the measurements. All steps following the addition of respiration buffers were performed rapidly to ensure consistency across measurements. We used two types of MiR05 negative controls: one including 5 μl of MitoXpress™ Xtra (Agilent Technologies, Santa Clara, CA, USA) and one with only MiR05. Our interplate control consisted of a mixture of 40 μl haemoglobin and MiR05. This protocol was repeated for each assay temperature used within a given experiment.

Respiration measurements were conducted for each test using a black, non-sterile, 96-well polystyrene plate with a transparent bottom (Greiner Bio-One GmbH, Kremsmünster, Austria). We pre-warmed the plate reader to the relevant assay temperature to ensure a stable temperature in the chamber throughout the measurements. The plates were read using bottom optics for 1 h and 30 min, with readings taken every 1 min and 30 s. Shaking was set to occur at 100 rpm in double orbital mode before each cycle.

Fluorescence signals were recorded as relative fluorescence units. The respiration rate for each well was determined by calculating slopes over sliding windows of four consecutive time points (with a total duration of ∼6 min) and selecting the maximum slope across all windows as the rate for that well. This approach captures peak respiration during the most stable phase of the signal and avoids the inclusion of later time points, which may reflect a decline in preparation quality. The 90-min measurement window was chosen to ensure all samples, including those with lower respiration rates, had sufficient time to reach a stable linear phase, rather than because the full duration was used for analysis. As a consequence, for strong respirations like OXPHOS, the slope was usually selected over four cycles within 30 min of the initiation of the measurement, whereas LEAK slopes were usually selected over four cycles within 40 min of the initiation of the run. Supplementary material showing representative fluorescence curves across the full assay period, with the selected analysis window indicated, is provided to allow evaluation of signal stability over time. A complete and detailed protocol, including supplementary calculation sheets in Excel and example R script for data organisation and calculation, is available in the supplementary materials.

#### Quality check of mitochondrial respiration during plate-based measurements

To evaluate whether the proposed plate-based protocol can disrupt mitochondrial integrity, we conducted an additional validation experiment involving 12 Japanese quails that were not included in Experiments 1 or 2. We followed the blood sample preparation and plate-reader protocol described above for Experiment 1, with samples measured at 36°C, 40°C, and 44°C. However, for each individual and temperature, a subset of wells used to measure OXPHOS_CI+CII_ additionally received exogenous cytochrome c (10 μM final concentration per well). Cytochrome c normally resides in the mitochondrial intermembrane space, where it transfers electrons from Complex III to Complex IV. If the outer mitochondrial membrane becomes permeable during sample preparation or measurement, endogenous cytochrome c can escape the intermembrane space. Under these conditions, exogenously added cytochrome c can access the electron transport chain and restore electron transfer. Consequently, a meaningful increase in OXPHOS respiration following the addition of cytochrome c indicates impaired mitochondrial integrity, whereas minimal change supports adequate mitochondrial integrity and respiration. Following HRR convention, we considered a percentage difference in OXPHOS exceeding 10%, with versus without cytochrome c, to be indicative of compromised outer membrane integrity (Kuznetsov et al., 2008; Perry et al., 2013; Kuang et al., 2022).

### Mitochondrial traits and their standardisation

We quantified the cell count using a CytoSMART Exact FL fluorescence cell counter (Axion BioSystems, Atlanta, GA, USA). For these analyses, 20 μl of blood was diluted 25-fold in PBS (Sigma-Aldrich, St. Louis, MO, USA). Wherever possible, we included cell count as a covariate in our analyses (see the ‘Statistical analysis’ section), rather than dividing respiration by cell count, except for the correlation, as recommended by Thoral et al. (2024). It should be noted that analyses not accounting for cell count yielded similar results and conclusions for both methods.

### Data cleaning

Prior to statistical analysis, we applied the following criteria for data selection, identically for both experiments. For Experiment 1, the initial sample size was 57. First, we removed all HRR measurements that showed clearly weak respiration (i.e. due to the residual presence of inhibitors from previous runs despite numerous washing steps; *n*=7), as well as every negative slope from the plate-reader data after accounting for non-mitochondrial respiration (*n*_OXPHOS_=3, *n*_LEAK_=12). For Experiment 2, the initial sample size was 19. Using the same criteria, we removed four negative plate-reader slopes for LEAK respiration after accounting for non-mitochondrial respiration to achieve a final sample size of 15.

Then, after calculation, OxCE was used to assess control efficiency. OxCE values near zero indicate essentially zero respiratory phosphorylation capacity (i.e. respiration dominated by leak/background error). In our HRR dataset, the average OxCE value was 0.82 (range 0.69–0.89), which is consistent with the results of avian blood studies that reported mitochondrial control efficiencies of around 0.8 (e.g. in permeabilised avian blood cells; Ton et al., 2025: [0.50–0.88]; Correia et al., 2025: [0.50–0.92]). No HRR measurements were excluded on the basis of OxCE. Plate-based fluorescence assays, however, do not yield comparable absolute OxCE values to HRR due to intrinsic methodological differences (Zdrazilova et al., 2022; Divakaruni and Jastroch, 2022; Gerencser et al., 2009). A threshold of OxCE>0.8, appropriate for HRR, is therefore not perfectly transferable to plate-based measurements. We therefore applied a fixed threshold of OxCE>0.50 to the plate-reader data only, set with reference to the distribution of values observed in our dataset, and based on our own HRR values and the HRR literature (Ton et al., 2025; Correia et al., 2025).

Data cleaning for both methods led to a final sample size of *n*_OXPHOS_=47, *n*_LEAK_=38, and *n*_OxCE_=24 for Experiment 1 and of *n*_OXPHOS_=19, *n*_LEAK_=15, and *n*_OxCE_=16 for Experiment 2. Since our objective was to test whether results obtained from both methods were similar, we restricted our analyses to the exact same dataset for both methods (i.e. minimum shared sample sizes stated above). For Experiment 1, results based on the maximum sample sizes available for each method and respiration rate are presented in the supplementary materials.

### Statistical analysis

To demonstrate the usefulness and validity of our plate-based approach for measuring mitochondrial aerobic metabolism in avian blood cells, we analysed the data using three complementary methods. First, we assessed the technical repeatability (*R*) and the associated 95% CIs of the raw OXPHOS and LEAK measurements obtained with the plate reader using the *rptR* package (Stoffel et al., 2017). To estimate the repeatability of OxCE from the plate-reader data, we arbitrarily assigned an identifier to each replicate of OXPHOS and LEAK (replicate 1 and replicate 2) and then computed OxCE for the combination of replicates 1 and 2. Repeatability was considered significant when the estimate and its 95% CI did not include zero. We did not estimate repeatability for the HRR method in the present experiment due to the limited blood volume and time constraints associated with the low-throughput nature of HRR. However, previous studies using HRR on avian blood cells have reported high repeatability of OXPHOS_CI+CII_ and LEAK_CI+CII_ measures (i.e. *R*_OXPHOS_ and *R*_LEAK_>0.90; Stier et al., 2017: *R*_OXPHOS_=0.95, *R*_LEAK_=0.91; Cossin-Sevrin et al., 2022: *R*_OXPHOS_=0.99, *R*_LEAK_=0.98; Cossin-Sevrin et al., 2023: *R*_OXPHOS_=0.98, *R*_LEAK_=0.98; Nord et al., 2023: *R*_OXPHOS_=0.95, *R*_LEAK_=0.96), while one study on fish blood cells presented a markedly lower repeatability (i.e. *R*_OXPHOS_=0.57 and *R*_LEAK_=0.45; Jégo et al., 2024).

Second, to assess agreement between the plate-reader and HRR methods, we quantified linear associations between OXPHOS_CI+CII_, LEAK_CI+CII_ and OxCE measured by each method using the Pearson's product-moment correlation coefficient. We computed Pearson's r with associated 95% CIs and two-tailed *P*-values for each trait, after standardising measurements by dividing by cell count and *z*-transforming variables to improve comparability of effect sizes using the *cor.test* function from the *stats* package in R (R Core Team, 2026, https://www.R-project.org/). The assumptions of Pearson's correlation (approximate bivariate normality and linear relationships) were checked visually using scatterplots and did not indicate strong deviations. For the correlation plots, we overlaid simple linear regression lines fitted by ordinary least squares to the same plate-reader versus HRR data that was used for Pearson's correlations and reported the corresponding Pearson's r and 95% CIs in the panel subtitles, using base functions from the *stats* package in R (R Core Team, 2026, https://www.R-project.org/). This was performed for both Experiments 1 and 2 separately, as well as together, for the measurements at 40°C.

Finally, to assess whether the two methods produced comparable results and led to similar biological conclusions, we analysed the data from the plate reader and HRR separately using linear mixed-effect models with identical fixed-effect structures and method-specific random-effect structures (Bates et al., 2015). For Experiment 1, fixed effects included the interaction between temperature and species (both treated as factors) and cell count as a covariate, except in models analysing OxCE (i.e. ratios derived from raw measurements). For the plate-reader data, plate ID (plate_temperature) was included as a random effect, whereas the combination of Oroboros ID-chamber ID was included as a random effect for the HRR data, in both cases together with sample ID as a random effect to account for repeated measurements of the same sample at the different temperatures. For Experiment 2, fixed effects included sex (female or male) and cell count as a covariate. Random-effect structure followed the same pattern as for Experiment 1, with the exception of sample ID, as each individual was accounted for only once. OXPHOS_CI+CII_ and LEAK_CI+CII_ variables were z-standardised prior to analysis to facilitate model convergence and interpretation of effect sizes. For each respiratory trait and method, we fitted linear mixed-effect models using the *lme4* package (Bates et al., 2015) and obtained χ^2^ and *P*-values for fixed effects from Type III Wald χ^2^ tests implemented in the *Anova* function of the *car* package (Fox and Weisberg, 2019).

For Experiment 1, we compared the amount of unexplained variance captured by each method. To do so, we performed F-tests on the residuals of paired models (plate reader versus HRR) for each respiratory state using the *var.test* function from the *stats* package in R (R Core Team, 2026, https://www.R-project.org/); an F-ratio of 1 indicates equal residual variance between methods, whereas values departing from 1 indicate that one method exhibits greater residual variance than the other. To further disentangle species- and temperature-specific responses, we repeated the linear mixed-effect models separately for each method and species. In these models, cell count was again included as a covariate when analysing OXPHOS_CI+CII_ and LEAK_CI+CII_, but not OxCE. Species-specific models derived from the *Experiment 1* dataset were also fitted using the maximum sample size available for each method and respiration; the corresponding results are presented in the supplementary materials (Tables S7 and S8).

To test whether the proportion of individual×temperature comparisons exceeding the 10% membrane-integrity threshold differed across assay temperatures in the quality-check validation experiment, we performed a chi-square test of independence on the corresponding contingency table, using the *chisq.test* function from the *stats* package in R (R Core Team, 2026, https://www.R-project.org/).

All statistical analyses were performed in R v.4.5.3 (R Core Team, 2026, https://www.R-project.org/). Model assumptions of normality and homoscedasticity were evaluated by inspecting diagnostic plots of residuals and fitted values. Statistical significance was defined as estimates with 95% CIs not overlapping zero or *P*≤0.05.

## Supplementary Material



10.1242/biolopen.062712_sup1 Supplementary information
