# Supplementary material for: High-throughput mitochondrial aerobic metabolism measurement: assessing a plate-reader approach using avian blood cells
Source: Biol Open. 2026 Oct 19;15(10):bio062712. doi: 10.1242/bio.062712 (PMC13730128; doi:10.1242/bio.062712)
Supplement: Supplementary file 1 [file biolopen-15-062712-s1.pdf]

Supplementary Materials and Methods

Raw data values of mitochondrial traits using both methods

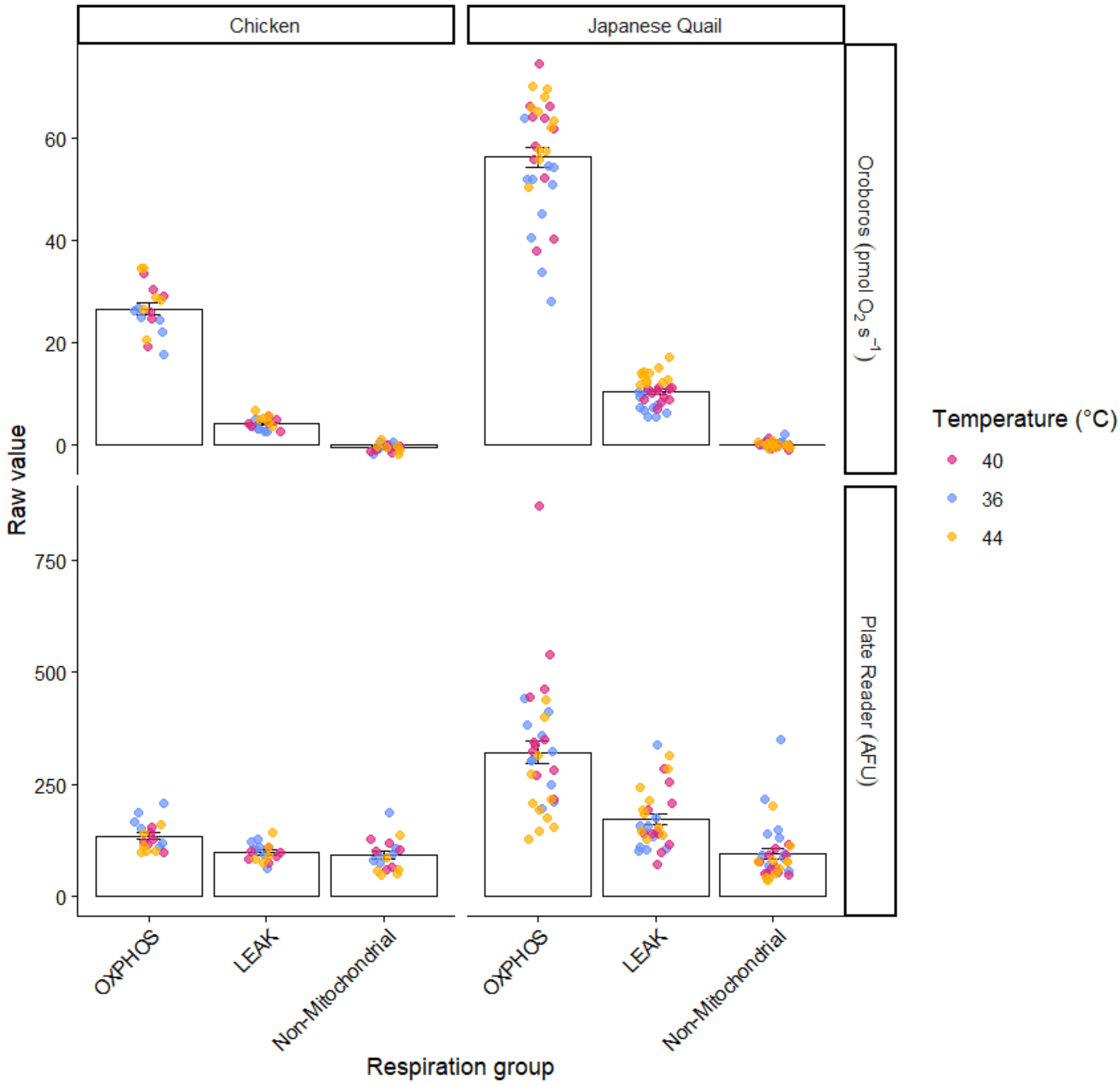

Fig. S1. Experiment 1: Mitochondrial respiration traits of chicken and Japanese quail whole blood measured in an Oroboros Oxygraph-2k and a fluorescence plate reader across three assay temperatures (36°C, 40°C, and 44°C). Bars show means ± s.e. for $OXPHOS_{CI+CII}$, $LEAK_{CI+CII}$, and non-mitochondrial respiration, with individual points representing raw values for each sample coloured by assay temperature. Plate reader raw values are expressed in relative fluorescent units (RFU).

Biology Open • Supplementary information

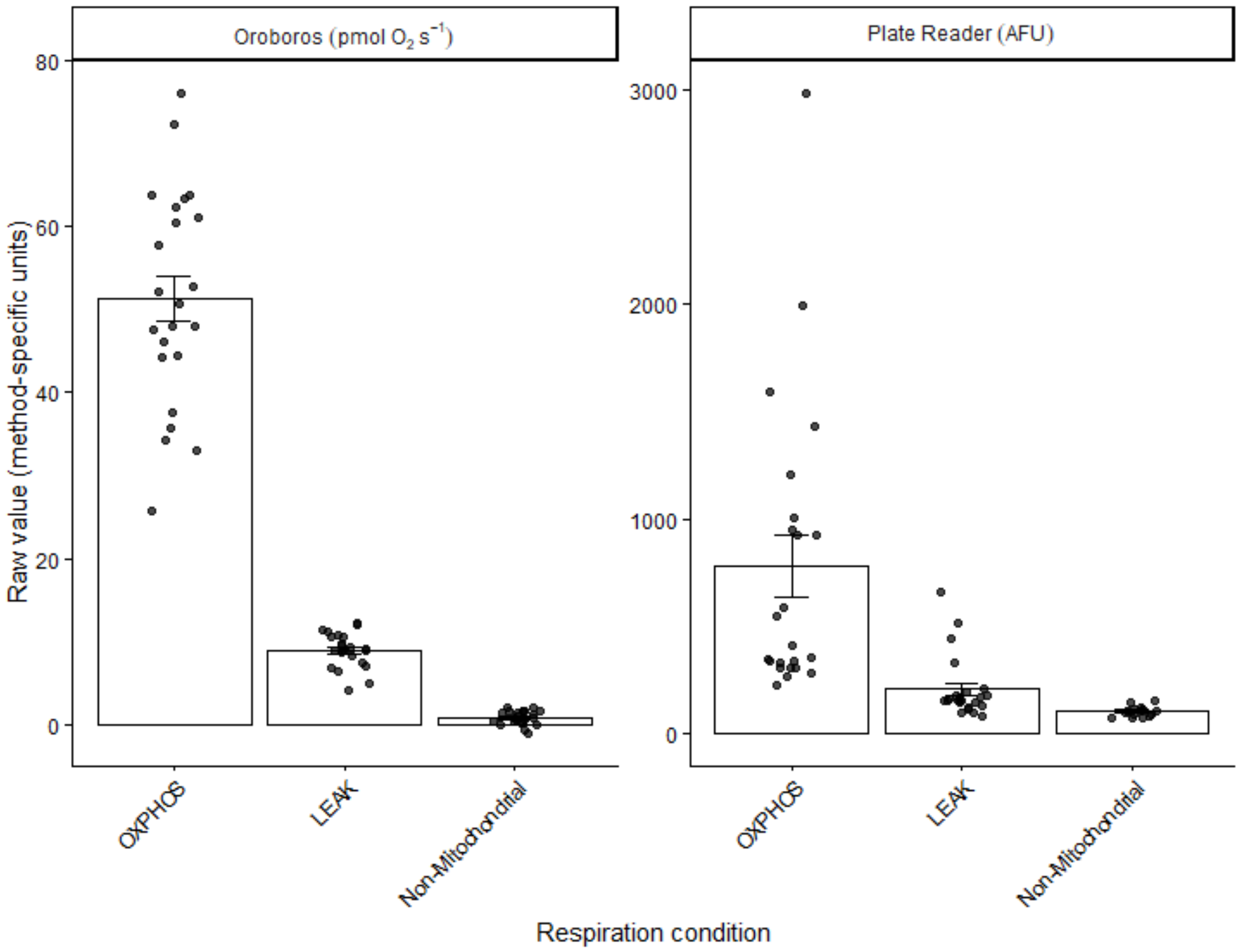

Fig. S2. Experiment 2: Mitochondrial respiration traits Japanese quail whole blood measured in an Oroboros Oxygraph-2k and a fluorescence plate reader. Bars show means ± s.e. for $OXPHOS_{CI+CII}$, LEAK $_{CI+CII}$, and non-mitochondrial respiration, with individual points representing raw values for each sample. Plate reader raw values are expressed in relative fluorescent units (RFU).

Biology Open • Supplementary information

Reagents and Equipment used in the present experimental study

Table S1. Mitochondrial Respiration Medium (Mir05) homemade reagents and CAS numbers provided by Oroboros Instruments (Fasching et al., 2016)

Provider	CAS Number	Name
**Sigma-Aldrich**	67-42-5	EGTA
**Sharlau**	7791-18-6	MgCl2.6 H2O
**Sigma-Aldrich**	96-82-2	Lactobionic acid
**Sigma-Aldrich**	107-35-7	Taurine
**Sigma-Aldrich**	7778-77-0	KH2PO4
**Sigma-Aldrich**	7365-45-9	HEPES
**Sigma-Aldrich**	57-50-1	D-Sucrose
**Sigma-Aldrich**	9048-46-8	BSA, essentially fatty acid free

Table S2. Respiration Buffer Reagents and CAS numbers used in the present analysis.

Provider	CAS Number	Name
**Sigma-Aldrich**	11024-24-1	Digitonin
**Sigma-Aldrich**	113-24-6	Pyruvic acid sodium salt
**Sigma-Aldrich**	1404-19-9	Oligomycin from Streptomyces diastatochromogenes
**Sigma-Aldrich**	1397-94-0	Antimycin A from Streptomyces sp.
**Sigma-Aldrich**	72696-48-1	Adenosine 5′-diphosphate monopotassium salt dihydrate
**Sigma-Aldrich**	97-67-6	L-Malic acid
**Sigma-Aldrich**	6106-21-4	Succinate disodium salt, hexahydrate
**Sigma-Aldrich**	67-68-5	DMSO
**Sigma-Aldrich**	9007-43-6	Cytochrome c
**Merck**	64-17-5	Ethanol (100%)
**Agilent**	MX-200-4	MitoXpress Xtra + Mineral Oil Packs
**Milian**	655096	Greiner Bio-One 96-well non-sterile polystyrene plate, colour plate: black with transparent bottom

Table S3. Instruments used in the present analysis

Name	Remarks and/or Recommendations
**Plate reader**	VANTAstar microplate reader (BMG Labtech, Ortenberg, Germany)
**Plate reader filter**	Excitation filter (Ex TR), Dichroic (LP TR), and Emission filter 645-20nm
**Oxygraph 2K high-resolution respirometer**	Oroboros Instruments (Innsbruck, Austria)

Detail of Pearson correlations coefficients from results section *"Correlations between the plate reader and HRR methods"*

Table S4. Pearson correlation coefficients between HRR methods and plate reader measurements of mitochondrial respiratory traits (OXPHOS, LEAK, OxCE) across temperatures (36°C, 40°C and 44°C), for both species together and separated. Values represent Pearson's *r* with 95% confidence intervals (CI). When NAs are displayed it reflect an insufficient sample size for correlation or interval estimation. For this reason, we also removed the OxCE section for chicken.

	Trait	Temperature (°C)	Pearson r [95% CI]	*n*
All species	OXPHOS	36	0.66 [0.23; 0.88]	15
		40	0.88 [0.67; 0.96]	16
		44	0.53 [0.05; 0.81]	16
	LEAK	36	-0.07 [-0.64; 0.55]	11
		40	0.32 [-0.31; 0.76]	12
		44	0.74 [0.37; 0.91]	15
	OxCE	36	0.32 [-0.34; 0.77]	11
		40	0.24 [-0.56; 0.81]	8
		44	0.64 [-0.56; 0.97]	5
Japanese quail	OXPHOS	36	0.44 [-0.30; 0.85]	9
		40	0.80 [0.40; 0.95]	11
		44	0.43 [-0.23; 0.81]	11
	LEAK	36	-0.41 [-0.89; 0.50]	7
		40	0.14 [-0.54; 0.70]	10
		44	0.70 [0.12; 0.92]	10
	OxCE	36	0.26 [-0.54; 0.81]	8
		40	0.42 [-0.48; 0.89]	7
		44	NA [NA]	2
Chicken	OXPHOS	36	-0.80 [-0.97; 0.04]	6
		40	0.18 [-0.83; 0.91]	5
		44	-0.09 [-0.90; 0.86]	5
	LEAK	36	0.94 [-0.20; 0.99]	4
		40	NA [NA]	2
		44	0.06 [-0.86; 0.89]	5

Biology Open • Supplementary information

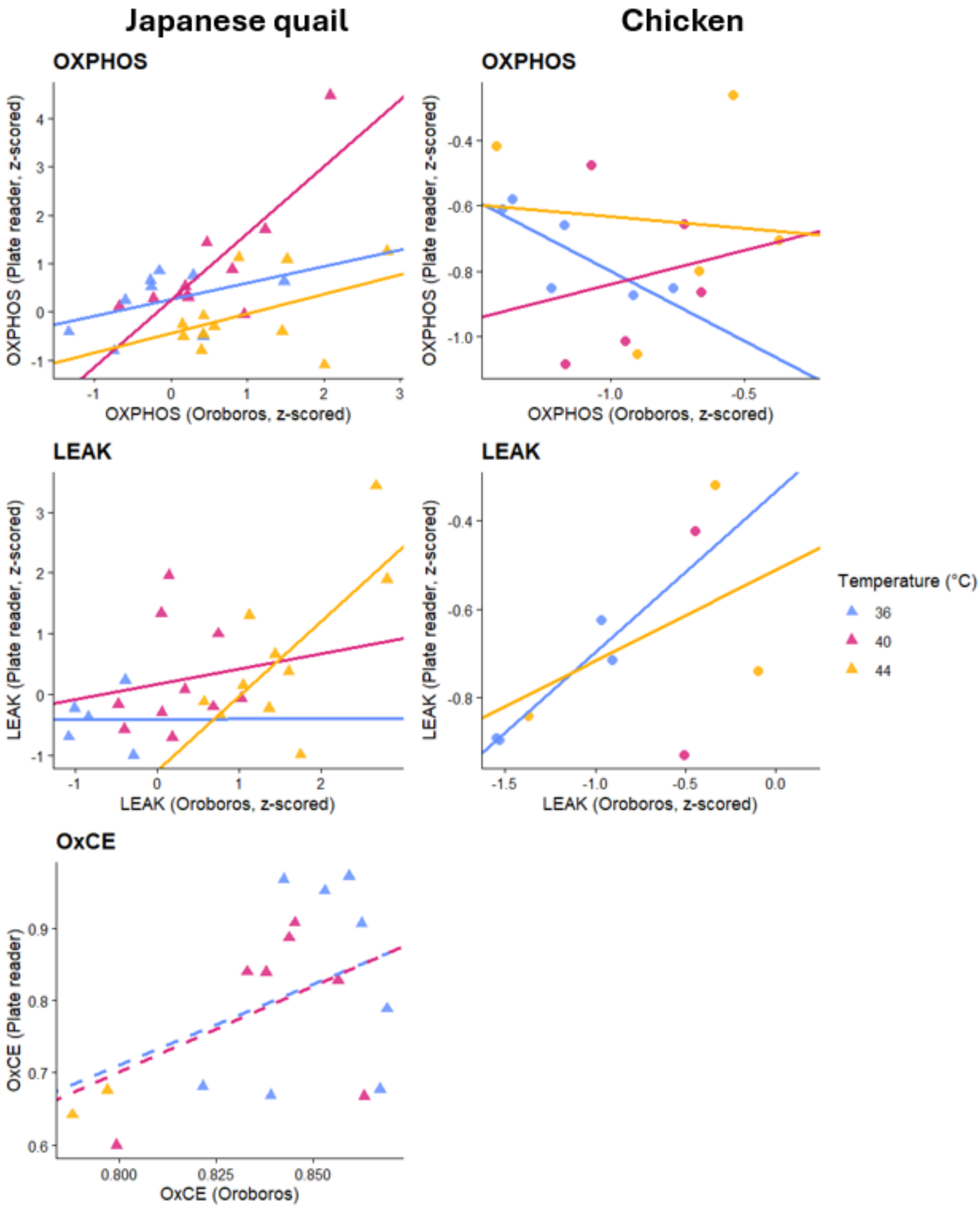

Fig. S3. Correlation between plate reader and high-resolution respirometry (Oroboros) measurements of $OXPHOS_{CI+CII}$ (top), $LEAK_{CI+CII}$ (middle), and OxCE (bottom), for Japanese quail (left) and chicken (right). Each point represents one individual, coloured by assay temperature (36°C, 40°C, 44° C), with a linear regression line fitted separately per temperature. All axes are z-scored except OxCE, which is shown on its raw scale. No OxCE panel is shown for chicken due to insufficient sample size to reliably estimate the plate reader–Oroboros relationship for this trait in this species.

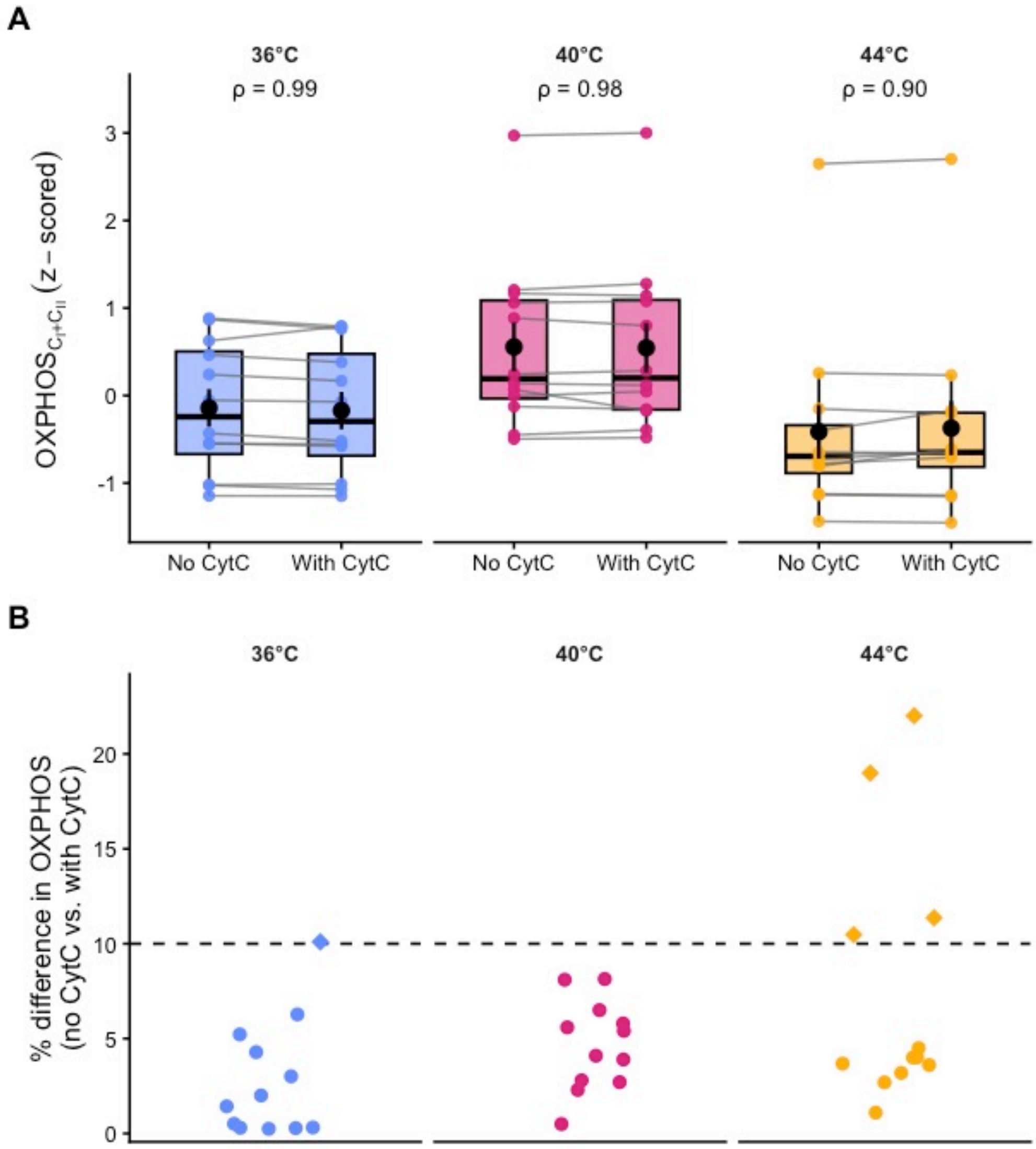

Fig. S4. Quality check of outer mitochondrial membrane integrity and retention of cytochrome c by measuring $OXPHOS_{CI+CII}$ respiration in the same samples without or with 10 µM of cytochrome c per well (n = 12 individuals per temperature). Panel A: $OXPHOS_{CI+CII}$ with vs. without cytochrome c for each individual, faceted by temperature; grey lines connect paired values from the same individual, black points and error bars show the mean ± SE for each condition (36°C: 129.7 ± 15.1 vs. 132.7 ± 15.4; 40°C: 178.4 ± 19.6 vs. 184.2 ± 20.5; 44°C: 110.7 ± 21.5 vs. 118.3 ± 22.3; no CytC vs. with CytC, respectively), and ρ is Spearman's rank correlation between the paired values at that temperature. Axes are z-scored to allow visual comparison across temperature panels. Panel B: percent difference in $OXPHOS_{CI+CII}$ with vs. without cytochrome c for each individual, relative to a pre-specified 10% threshold for membrane compromise (dashed line); diamonds mark comparisons exceeding this threshold. 5/36 individual×temperature comparisons (13.9%) exceeded the threshold overall (36°C: 1/12; 40°C: 0/12; 44°C: 4/12).

Detail of models estimates from results section *“Comparison of the plate reader and high resolution respirometry measurements in two species across various temperatures”*

Table S5. Detailed results from linear mixed-effects models evaluating the effects of interassay temperature (40°C vs. 36°C and 44°C) and cell count as fixed covariates, including model estimates, standard errors (SE), degrees of freedom (df), and p-values, for chicken mitochondrial respiration parameters: $OXPHOS_{CI+CII}$, $LEAK_{CI+CII}$, and OxCE measured by plate reader vs. high-resolution respirometry. The data have been scaled for the analyses, enabling comparisons between estimates from the two methods.

		Plate reader					High resolution respirometry				
		Estimate	SE	df	t	p-value	Estimate	SE	df	t	p-value
**$OXPHOS_{CI+CII}$**	(Intercept)	-1.22	1.55	12.0	-0.79	0.445	**-2.55**	**0.83**	**12.0**	**-3.06**	**0.010**
N = 6<br>n = 16	Temperature36	0.49	0.84	12.0	0.59	0.568	**-0.92**	**0.36**	**12.0**	**-2.58**	**0.024**
	Temperature44	0.75	0.85	12.0	0.88	0.398	0.31	0.37	12.0	0.83	0.423
	Cell count	0.01	0.02	12.0	0.58	0.572	**0.05**	**0.01**	**12.0**	**3.63**	**0.003**
**$LEAK_{CI+CII}$**	(Intercept)	-1.05	2.15	5.1	-0.49	0.645	-0.31	1.64	7.0	-0.18	0.856
N = 5<br>n = 11	Temperature36	-0.21	0.95	4.3	-0.23	0.831	-1.42	0.82	7.0	-1.73	0.126
	Temperature44	-0.38	0.90	5.1	-0.42	0.689	-0.15	0.77	7.0	-0.20	0.845
	Cell count	0.02	0.03	5.3	0.61	0.566	0.01	0.02	7.0	0.56	0.593
**OxCE**	(Intercept)	1.19	1.05	4.0	1.13	0.319	-1.25	0.90	4.0	-1.37	0.240
N = 7<br>n = 7	Temperature36	-1.32	1.22	4.0	-1.08	0.338	1.84	1.04	4.0	1.76	0.153
	Temperature44	-1.45	1.22	4.0	-1.19	0.298	1.07	1.04	4.0	1.02	0.364

Note: p-values <0.05 in **bold** (two-tailed t-tests). N=individuals, n=observations.

Biology Open • Supplementary information

Table S6. Detailed results from linear mixed-effects models evaluating the effects of interassay temperature (40°C vs. 36°C and 44°C) and cell count as fixed covariates, including model estimates, standard errors (SE), degrees of freedom (df), and p-values, for Japanese quail mitochondrial respiration parameters: $OXPHOS_{CI+CII}$, $LEAK_{CI+CII}$, and OxCE measured by plate reader vs. high-resolution respirometry. The data have been scaled for the analyses, enabling comparisons between estimates from the two methods.

		Plate reader					High resolution respirometry				
		Estimate	SE	df	t	p-value	Estimate	SE	df	t	p-value
**$OXPHOS_{CI+CII}$**	(Intercept)	0.30	1.80	8.4	0.17	0.872	-0.97	1.51	9.2	-0.65	0.535
N = 13<br>n = 31	Temperature36	-0.60	0.41	1.7	-1.46	0.300	**-0.97**	**0.29**	**15.0**	**-3.33**	**0.005**
	Temperature44	-1.07	0.39	1.5	-2.74	0.156	0.30	0.27	16.1	1.13	0.277
	Cell count	0.00	0.02	8.0	0.13	0.899	0.01	0.02	7.9	0.75	0.474
**$LEAK_{CI+CII}$**	(Intercept)	2.50	1.32	23.0	1.90	0.070	-1.04	0.88	9.7	-1.18	0.266
N = 13<br>n = 27	Temperature36	**-0.97**	**0.43**	**23.0**	**-2.27**	**0.032**	**-0.86**	**0.16**	**14.3**	**-5.42**	**<0.001**
	Temperature44	0.10	0.40	23.0	0.24	0.811	**1.30**	**0.13**	**12.3**	**9.63**	**<0.001**
	Cell count	-0.03	0.02	23.0	-1.82	0.081	0.01	0.01	8.5	0.86	0.415
**OxCE**	(Intercept)	0.01	0.36	14	0.03	0.977	-0.05	0.26	9.57	-0.216	0.833
N = 12<br>n = 17	Temperature36	0.24	0.50	14	0.48	0.633	0.63	0.28	5.61	2.227	0.070
	Temperature44	-1.074	0.77	14	-1.38	0.189	**-1.77**	**0.39**	**2.70**	**-4.525**	**0.025**

Note: p-values <0.05 in **bold** (two-tailed t-tests). N = individuals, n = observations.

Table S7. Detailed results from linear mixed-effects models evaluating the effects of interassay temperature (40°C vs. 36°C and 44°C) and cell count as fixed covariates, including model estimates, standard errors (SE), degrees of freedom (df), and p-values, for chicken mitochondrial respiration parameters, on complete sample size per methods: $OXPHOS_{CI+CII}$, $LEAK_{CI+CII}$, and OxCE measured by plate reader vs. high-resolution respirometry. The data have been scaled for the analyses, enabling comparisons between estimates from the two methods.

		Plate reader					High resolution respirometry				
		Estimate	SE	df	t	p-value	Estimate	SE	df	t	p-value
**$OXPHOS_{CI+CII}$**	(Intercept)	-1.22	1.55	12.0	-0.79	0.445	**-2.88**	**0.65**	**4.61**	**-4.45**	**0.008**
$N_{Plate\ reader}$ = 6<br>$n_{Plate\ reader}$ = 16	Temperature36	0.49	0.84	12.0	0.59	0.568	**-0.77**	**0.30**	**10.0**	**-2.61**	**0.026**
N_{HRR} = 6<br>n_{HRR} = 18	Temperature44	0.75	0.85	12.0	0.88	0.398	0.28	0.30	10.0	0.94	0.371
	Cell count	0.01	0.02	12.0	0.58	0.572	**0.05**	**0.01**	**4.00**	**5.01**	**0.007**
**$LEAK_{CI+CII}$**	(Intercept)	-1.05	2.15	5.1	-0.49	0.645	-1.29	0.82	4.82	-1.55	0.18
$N_{Plate\ reader}$ = 5<br>$n_{Plate\ reader}$ = 11	Temperature36	-0.21	0.95	4.3	-0.23	0.831	**-1.05**	**0.43**	**10.0**	**-2.43**	**0.03**
N_{HRR} = 6<br>n_{HRR} = 18	Temperature44	-0.38	0.90	5.1	-0.42	0.689	0.39	0.43	10.0	0.89	0.39
	Cell count	0.02	0.03	5.3	0.61	0.566	0.02	0.01	4.0	1.96	0.12
**OxCE**	(Intercept)	1.19	1.05	4	1.13	0.319	-0.23	0.37	14.3	-0.62	0.543
$N_{Plate\ reader}$ = 7<br>$n_{Plate\ reader}$ = 7	Temperature36	-1.32	1.22	4	-1.08	0.338	0.92	0.48	10	1.89	0.087
N_{HRR} = 6<br>n_{HRR} = 18	Temperature44	-1.45	1.22	4	-1.19	0.298	-0.22	0.48	10	-0.46	0.652

Note: p-values <0.05 in **bold** (two-tailed t-tests). N=individuals, n=observations.

Table S8. Detailed results from linear mixed-effects models evaluating the effects of interassay temperature (40°C vs. 36°C and 44°C) and cell count as fixed covariates, including model estimates, standard errors (SE), degrees of freedom (df), and p-values, for Japanese quail mitochondrial respiration parameters, on complete sample size per methods: $OXPHOS_{CI+CII}$, $LEAK_{CI+CII}$, and OxCE measured by plate reader vs. high-resolution respirometry. The data have been scaled for the analyses, enabling comparisons between estimates from the two methods.

		Plate reader					High resolution respirometry				
		Estimate	SE	df	t	p-value	Estimate	SE	df	t	p-value
**$OXPHOS_{CI+CII}$**	(Intercept)	0.23	1.77	11.54	0.13	0.896	-0.81	1.52	8.61	-0.53	0.60
$N_{Plate\ reader} = 13$<br>$n_{Plate\ reader} = 38$	Temperature36	-0.51	0.36	1.58	-1.40	0.327	**-0.87**	**0.29**	**14.6**	**-3.03**	**0.008**
$N_{HRR} = 13$<br>$n_{HRR} = 32$	Temperature44	-0.97	0.36	1.51	-2.70	0.152	0.30	0.27	16.6	1.09	0.287
	Cell count	0.002	0.02	11.45	0.12	0.902	0.01	0.01	7.36	0.63	0.542
**$LEAK_{CI+CII}$**	(Intercept)	1.42	1.17	30.0	1.21	0.235	-0.66	0.95	10.1	-0.69	0.50
$N_{Plate\ reader} = 13$<br>$n_{Plate\ reader} = 34$	Temperature36	**-0.79**	**0.39**	**30.0**	**-2.03**	**0.050**	**-0.68**	**0.14**	**17.6**	**-4.86**	**<0.001**
$N_{HRR} = 13$<br>$n_{HRR} = 32$	Temperature44	0.23	0.37	30.0	0.68	0.534	**1.30**	**0.13**	**15.9**	**9.92**	**<0.001**
	Cell count	-0.01	0.01	30.0	-1.10	0.277	0.005	0.01	9.2	0.45	0.661
**OxCE**	(Intercept)	-0.08	0.32	20	-0.26	0.796	0.29	0.23	16.8	1.25	0.228
$N_{Plate\ reader} = 13$<br>$n_{Plate\ reader} = 23$	Temperature36	0.36	0.44	20	0.83	0.413	0.35	0.31	19.1	1.13	0.270
$N_{HRR} = 13$<br>$n_{HRR} = 32$	Temperature44	-0.69	0.65	20	-1.06	0.300	**-1.20**	**0.30**	**17.6**	**-3.97**	**<0.001**

Note: p-values <0.05 in **bold** (two-tailed t-tests). N=individuals, n=observations.

Biology Open • Supplementary information
