# Supplementary material for: High-throughput mitochondrial aerobic metabolism measurement: assessing a plate-reader approach using avian blood cells
Source: Biol Open. 2026 Oct 19;15(10):bio062712. doi: 10.1242/bio.062712 (PMC13730128; doi:10.1242/bio.062712)
Supplement: Supplementary file 2 [file biolopen-15-062712_review_history.pdf]

High-throughput mitochondrial aerobic metabolism measurement: assessing a plate-reader approach using avian blood cells

Cloé Hadjadji, Molly Ohse, Antoine Stier and Pierre Bize

10.1242/bio.062712

Editor: Nicholas Wu

Review timeline

Original submission:	22 May 2026
Editorial decision:	2 June 2026
First revision received:	25 August 2026
Editorial decision:	31 August 2026
Second revision received:	18 September 2026
Accepted:	21 September 2026

Original submission

First decision letter

MS ID#: bio.062712

MS Title: High-throughput mitochondrial aerobic metabolism measurement: assessing a plate-reader approach using avian blood cells

Authors: Cloé Hadjadji; Molly Ohse; Antoine Stier; Pierre Bize

I have now reached a decision on the above manuscript.

The reviewer reports are shown at the bottom of this email.

As you will see, the reviewers raised a number of substantial criticisms that prevent me from accepting the paper at this stage. When the manuscript came to my attention, I was quite excited about the idea of a high-throughput plate reader approach to quantifying mitochondrial metabolism. Any techniques to make physiological approaches more accessible to a wider group, I am interested in. Both reviewers also liked the idea, but after reading through both reviewers' comments and checking the paper myself, there are a number of potentially serious methodological issues that prevent me from accepting the paper as it is. In order for the technique to be useful, it must balance between accuracy and convenience relative to gold standard approaches (e.g. Oroboros O2k), which reviewer 1 highlighted that the plate reader approach presented has a number of limitations that prevent it from being useful more broadly. If you are able to respond satisfactorily with the criticisms on revision, I would be pleased to see a revised manuscript. We would then return it to the reviewers.

At this stage, we also ask you to ensure your manuscript complies with our formatting guidelines.

Please upload both a 'clean' version of your Word file, along with a highlighted version clearly showing where you have made changes in the revised manuscript. Please avoid using 'Track changes' in Word files as these are lost in PDF conversion.

© 2026. Published by The Company of Biologists under the terms of the Creative Commons Attribution License (https://creativecommons.org/licenses/by/4.0/).

I should be grateful if you would also provide a point-by-point response detailing how you have dealt with the points raised by the reviewers in the 'Response to Reviewers' box. Please attend to all of the reviewers' comments. If you do not agree with any of their criticisms or suggestions please explain clearly why this is so.

Reviewer 1

Comments for the author

Overview: In the present manuscript, Hadjadji et al attempt to establish a fluorescence-based plate-reader high throughput assay of mitochondrial respiration by comparing it to the gold standard of high resolution respirometry (HRR) using avian red blood cells. Although the manuscript is quite well written and the authors have taken great pains in the analysis and normalization of their data, I find the data provides very poor support for the primary claims of the paper. In particular, the plate based assay, despite huge efforts to "clean" the data and look for relationships, has very low signal resolution, and provides a highly variable dataset that does not clearly recapitulate the "real" relationships between species and temperature revealed by the HRR approach. In the present paper the authors can identify this issue because they have used both approaches but in a future experiment where the plate-based assay was conducted only, the results would be impossible to interpret and relatively meaningless because there is clearly a very high likelihood of false negatives. If the authors wish to move forward with this publication then additional data that actually supports their conclusion is necessary (likely using far higher-throughput assays to drastically increase the sample replicate number). Alternatively, the authors could more accurately discuss their results and reach the conclusion that the plate-based assay is a poor representative of the true results obtainable from HRR. I offer the following specific comments for the authors to consider in revising their manuscript.

Major concerns:

1. The introduction is quite well-written and is generally very comprehensive and well-referenced. However, some statements and claims are a bit of an exaggeration:

a. In the intro, the authors discuss the repeatability limits of running duplicates in separate oroboros chambers but do not address the downsides of using a plate reader. For example, edge effects mean all wells may not necessarily be measured evenly and thus duplicates are not always straightforward without various controls, just as for the oroboros. I encourage the authors to present a more balanced presentation of the pros and cons of both approaches and not just the positives of the plate reader approach.

b. Although generally factually accurate, the introduction oversells the relationship of red blood cell mitochondrial function to tissue and whole animal metabolism. A careful look at the cited papers reveals that the correlation between RBC mitos and tissue mitos are either not very strong (< 0.7) or are more robust but with a very shallow slope that reveals very small changes in RBC mito respiration with larger changes in tissue respiration. The relationships are significantly correlated but the very low sensitivity of RBC assays relative to tissue assays suggests that the curve is very shallow and small, and thus has poor sensitivity to changes, which is fine for HRR approaches but likely contributes to very low resolution in the high throughput approach where tissues and assays are not as granular as with HRR. Similarly, the relationship between RBC respiration and whole animal metabolic rate correlated only with nighttime metabolism and not daytime metabolism, from which the cells were isolated…this suggests a bit of a contorted effort to squint and make a story work, rather than a robust correlation upon which to build the present study. I encourage the authors to present this introductory information in a more balanced fashion to reflect these less-than-ideal relationships as such.

2. The authors ignore existing products that achieve many of their aims. Isn't the Agilent Seahorse basically a plate reader that measures oxygen consumption in real time and at relatively high throughputs than HRR? What is novel about the current approach in terms of the technique and design? I recognize that this approach doesn't require the purchase of a Seahorse but it does require

© 2026. Published by The Company of Biologists under the terms of the Creative Commons Attribution License (https://creativecommons.org/licenses/by/4.0/).

the purchase of a plate reader, if one isn't already available. In either case, the current discussion of options available to researchers (HRR and the present approach) ignores this key existing technology. Please include discussion of where the seahorse fits into the field in the introduction to appropriately position the need for the present approach.

3. Methods: I am unclear regarding the workflow for blood collection and experimentation. The authors describe collecting blood and processing it within 2 hours but they also describe measuring the same blood with three different temperatures in assays that are each 90 minutes long on a single plate reader. Something doesn't add up here. Were samples used across the full ~ 5+ hours of time required to support these 3 different temperature runs on microplates? But then the authors say the assay was run across 3 days…so does this mean the same blood sample was kept on ice for 72 hours across these assays and tested each day? This would be concerning and evidence that the blood and mitochondria are still highly viable after 72 hours would be necessary. Overall, I found the description of tissue handling and workflow to be very hard to decipher.

4. Duration: 90 minutes is quite a long assay time for mitochondrial measurements and may lead to degeneration of the quality of the preparation. The authors indicate that they took measurements throughout this period but it is not clear what period was used for the final analysis….was it averaged across the full 90 minutes? It would be useful to see the rates across time with each 90 sec bin plotted so that the reader can evaluate how assay time is impacting the respiration rate of mitochondria in this environment.

5. Mito quality: There is a clear and very significant difference in OxCE between preparations, both in terms of the large difference between means in all species and conditions but also the large variability apparent in the plate spec assay relative to the very tight datasets for the HRR. Many of the values for OxCE are quite low and the threshold of 0.4 likely includes a lot of mitochondria that are in rough shape. Usually, it is ideal to see numbers of > 0.8 for healthy and reliable mitochondrial preparations. I think the huge differences in mean and variability between preparations clearly demonstrate that the plate assay is not recapitulating the data obtained from HRR and call into question the viability of the plate prep.

6. Temperature effects and sensitivity. The HRR data returned the expected relationship such that with increasing temperatures, respiration rate increases (and vice versa with colder temperatures). However, this is clearly not recapitulated in the plate reader data. There seems to be a total failure of the HSS assay to measure the impact of this experimental condition on mito respiration in RBCs in either species. If the plate based assay cannot detect temperature-dependent changes (which, from the HRR data are robust and clear) then this calls into question the ability of this assay to reliably detect biological changes in a wide variety of testable conditions…or species differences that are not as robust as happened to emerge between the two birds studied herein.

7. Data variability. The data is highly variable and the ability to detect changes is suspect. The authors note this themselves when describing how estimates "differed in magnitude and direction between methods and species." That is not a reliable assay! The authors conclude that the plate reader assay can be accurate under "specific assay conditions" but these conditions are not known unless HRR is conducted in parallel to fact-check the data of the plate reader. In this case the temperature data was not useful but some of the species comparisons were…but in a novel experiment a researcher wouldn't know what data was real and what was not without conducting the more time-consuming assay. As such, the chances of false positives and false negatives is very very high, which is not useful for plate-based high throughput assays. The authors conclude that this assay may not be good for looking at the effects of temperature but how would one know if it is effective at looking at the effects of other treatments without the same control approaches? The fact that some differences were detectable is really more about luck/chance and I don't think reflects widespread reliability in this method. How is a researcher to know what is a "real" change and what is a failure of the system to detect a change? The error rate will be enormous and significant and constantly provide researchers with incorrect information. The authors suggest that with higher sample numbers the values would average out and become more accurate but as the paper stands there is really very little data to support their conclusions, and the bulk of data suggests that the plate based assay is not at all appropriate for these research questions. If the authors wish to proceed with this claim then at the very least they must show that it works. Additional experiments with higher replicates (384 well plates) to have sufficient sample sizes to

© 2026. Published by The Company of Biologists under the terms of the Creative Commons Attribution License (https://creativecommons.org/licenses/by/4.0/).

reliably show the same relationships as HRR reveals is an absolute minimum requirement for publication of this data because otherwise there is no support for the authors present conclusions.

8. The cells spend a long time in the wells and may become hypoxic in that period. The high degree of variability and poor OxCE values suggest damaged and less functional mitochondria. The authors also suggest that cells at the higher temperature may have been exposed to heat stress before the assay began. Wouldn't this issue also spill over into other stress tests that researchers might potentially explore with this system? I would like to see some cell viability data for samples or other measures of mitochondrial viability (for cells treated IN the plate reader). Without quality controls, any mitochondrial results are relatively meaningless, and I fail to see any quality control built into the plate reader experiments at present, based on the criticism of the OxCE approach included in the Discussion section.

9. Overstating the data. The authors conclude that the plate assay captured broadly similar patterns to HRR. The data does not support this. There is far greater variability and no ability to detect temperature based changes accurately between species. The assay does detect broad patterns between species when temperature is not compared but these differences are generally pretty large, likely reflecting the very low power of the plate based assays….and a lot of chance that these species happened to have large enough differences to measure with this very crude approach.

Reviewer 2

Comments for the author

'High-throughput mitochondrial aerobic metabolism measurement: validating a plate-reader approach using avian blood cells ' is a well-written and well-structured manuscript. I appreciate the authors' effort in developing and validating a fluorescence-based microplate reader approach to assess mitochondrial aerobic metabolism, including oxidative phosphorylation (OXPHOS), leak respiration, and oxidative control efficiency (OxCE; the difference between OXPHOS and leak respiration), using blood samples from chickens and Japanese quail at three temperatures (36°C, 40°C, and 44°C). These temperatures represent hypothermic, normothermic, and hyperthermic conditions, respectively, in the study organisms.

The authors demonstrate that, under normothermic conditions, measurements of OXPHOS and leak respiration obtained using the microplate reader assay correlate well with those obtained using the widely accepted high-resolution respirometry (HRR) approach with the Oxygraph O2k. However, these correlations appear weaker at hypothermic and hyperthermic temperatures. In addition, oxidative control efficiency did not show a significant correlation between the two methods at any of the tested temperatures.

The development of a high-throughput method for estimating mitochondrial aerobic metabolism would represent a valuable advancement for the field. Such an approach could serve as an efficient screening tool prior to the application of more precise and labor-intensive HRR measurements. Therefore, this study has the potential to make a meaningful contribution to the mitochondrial physiology research community. Overall, the manuscript is comprehensive and thoughtfully discusses both the strengths and limitations of the proposed methodology.

I nevertheless have several suggestions that I believe would strengthen the manuscript prior to publication:

1. Lines 234-254 (Microplate-based fluorescent assay): As this is primarily a methods-focused manuscript, I recommend including the exact concentrations of all reagents used in the assay within the main text. The authors refer readers to the supplementary material for these details; however, I was unable to locate the complete reagent concentrations. Providing this information directly in the manuscript would improve reproducibility and accessibility.

2. Lines 209-212: The authors used antimycin A to assess non-mitochondrial respiration. However, inhibitors such as potassium cyanide (KCN) or sodium azide are more commonly used for this

© 2026. Published by The Company of Biologists under the terms of the Creative Commons Attribution License (https://creativecommons.org/licenses/by/4.0/).

purpose. I suggest that the authors provide a justification for their choice of antimycin A as the sole inhibitor and discuss any potential implications of this methodological decision.

3. Figure 2: I recommend including separate regression lines for each temperature treatment within each parameter panel (OXPHOS, leak respiration, and OxCE). Additionally, presenting the correlations separately for chickens and Japanese quail would improve transparency and provide a clearer assessment of assay performance across species and temperature conditions.

4. Discussion (Lines 473-475): The relationship between the microplate reader assay and HRR measurements may vary among species due to differences in blood cell physiology, mitochondrial characteristics, or thermal sensitivity. I encourage the authors to acknowledge this possibility and include species-specific validation as an important consideration for future studies.

Reviewer's Responses to Questions

Experimental quality
Are experiments performed using appropriate methods that will answer the question (or test the hypothesis or support the observations) posed by the authors? Is the right tool used for the job?
If 'No', please indicate reasons in Comments for Author box below.
Reviewer #1:

- No

Reviewer #2:

- Yes

Were the data analyzed using appropriate statistical tests?
If 'No', please indicate reasons in Comments for Author box below.
Reviewer #1:

- Yes

Reviewer #2:

- Yes

Reproducibility
Were experiments performed using adequate number of biological replicates?
If 'No', please indicate reasons in Comments for Author box below.
Reviewer #1:

- No

Reviewer #2:

- No

Is there sufficient raw data to assess the rigor of the analysis?
If 'No', please indicate reasons in Comments for Author box below.
Reviewer #1:

- Yes

Reviewer #2:

- Yes

Does the methods section provide sufficient detail to permit reproducibility?
If 'No', please indicate reasons in Comments for Author box below.
Reviewer #1:

- No

© 2026. Published by The Company of Biologists under the terms of the Creative Commons Attribution License (https://creativecommons.org/licenses/by/4.0/).

Reviewer #2:
- No

Completeness
Are the manuscript's conclusions supported by the data?
If 'No', please indicate reasons in Comments for Author box below.
Reviewer #1:
- No

Reviewer #2:
- Yes

Scholarship
Do the authors cite and discuss the merits of data that would argue for and against their conclusion?
If 'No', please indicate reasons in Comments for Author box below.
Reviewer #1:
- Yes

Reviewer #2:
- Yes

Does the manuscript title & abstract accurately reflect the contents of the manuscript, without hyperbole?
If 'No', please indicate reasons in Comments for Author box below.
Reviewer #1:
- No

Reviewer #2:
- Yes

First revision

Author response to reviewers' comments

Dear Editor,

We are pleased to submit the revised version of our paper initially entitled "High-throughput mitochondrial aerobic metabolism measurement: validating a plate-reader approach using avian blood cells" which we would like to see published in *Biology Open*. Please find hereafter the comments from Editors and Reviewers, and our responses. In bold are the comments of the reviewers and in normal font are our responses.

In response to concerns raised by Reviewer 1 regarding the robustness of our conclusions from a single, three-temperature experimental design, we have substantially revised the manuscript by adding a second, independent experiment. This new experiment compares the plate reader and high-resolution respirometry methods in Japanese quail under a single, near-physiological temperature (40°C), testing their agreement in detecting effects of sex. This addition allowed us to test the assay's reliability under simpler, well-controlled conditions, and led us to revise our conclusions throughout the manuscript to more precisely reflect where the plate-based assay performs reliably relative to HRR. The title of the revised version would now be: "High-throughput mitochondrial aerobic metabolism measurement: assessing a plate-reader approach using avian blood cells".

© 2026. Published by The Company of Biologists under the terms of the Creative Commons Attribution License (https://creativecommons.org/licenses/by/4.0/).

We thank the reviewers for their comments and suggestions, that we have followed carefully, as you will see below. We believe that reviewers' comments greatly improved our manuscript, and we hope that it is now acceptable for publication in *Biology Open*.

Thank you very much for considering our work.

With our best regards,

On behalf of co-authors of the revised version of the manuscript **bio.062712**, Cloé Hadjadji

Reviewer 1: Overview

In the present manuscript, Hadjadji et al attempt to establish a fluorescence-based plate-reader high throughput assay of mitochondrial respiration by comparing it to the gold standard of high resolution respirometry (HRR) using avian red blood cells. Although the manuscript is quite well written and the authors have taken great pains in the analysis and normalization of their data, I find the data provides very poor support for the primary claims of the paper. In particular, the plate based assay, despite huge efforts to "clean" the data and look for relationships, has very low signal resolution, and provides a highly variable dataset that does not clearly recapitulate the "real" relationships between species and temperature revealed by the HRR approach. In the present paper the authors can identify this issue because they have used both approaches but in a future experiment where the plate-based assay was conducted only, the results would be impossible to interpret and relatively meaningless because there is clearly a very high likelihood of false negatives. If the authors wish to move forward with this publication then additional data that actually supports their conclusion is necessary (likely using far higher-throughput assays to drastically increase the sample replicate number). Alternatively, the authors could more accurately discuss their results and reach the conclusion that the plate-based assay is a poor representative of the true results obtainable from HRR. I offer the following specific comments for the authors to consider in revising their manuscript.

We thank the reviewer for their time and constructive comments, which have substantially improved the manuscript. We also thank the reviewer for acknowledging the effort we put into analysing and cleaning the data, along with writing. We acknowledge that the strength of our conclusions was maybe insufficiently supported by the data we presented. In response, we have chosen a combined approach: we added new data to strengthen the method comparison with a within-species comparison under normothermic conditions (i.e. differences between sexes in quail), and we have revised our conclusions to more accurately reflect the limits of the plate-based assay relative to HRR, to better avoid overstating its performance. The main conclusion is that the plate reader seems to perform relatively well to detect: 1. large effect size (i.e. species effect across temperatures for both LEAK and OXPHOS), 2. more subtle effects under normothermic condition and sufficiently high respiratory fluxes (i.e. sex effect on OxCE in quail, with comparable effect sizes for OXPHOS though only reaching formal significance with HRR). The full manuscript has also been updated accordingly, especially the method section. In addition, a planned study from our group will include an HRR cross validation in the Alpine swift, as it will be the first study using a primarily plate-based approach.

Reviewer 1: Specific Comments

---

1. The introduction is quite well-written and is generally very comprehensive and well-referenced. However, some statements and claims are a bit of an exaggeration:

© 2026. Published by The Company of Biologists under the terms of the Creative Commons Attribution License (https://creativecommons.org/licenses/by/4.0/).

a. In the intro, the authors discuss the repeatability limits of running duplicates in separate oroboros chambers but do not address the downsides of using a plate reader. For example, edge effects mean all wells may not necessarily be measured evenly and thus duplicates are not always straightforward without various controls, just as for the oroboros. I encourage the authors to present a more balanced presentation of the pros and cons of both approaches and not just the positives of the plate reader approach.

We agree that a more balanced treatment of the two methods' limitations is merited. We have revised the sentence in the Introduction contrasting duplicate measurements between the two methods, removing the reference to plate-reader duplicates as 'true duplicates,' as this wording implied an equivalence across wells that is not necessarily the case without further analysis (Line 562). We have also incorporated an explicit discussion of edge effects and the need for positional controls in the plate- based approach; on reflection, we felt this point was better placed in the Discussion, alongside our technical repeatability results, where it can be interpreted in light of the data obtained, rather than introduced as an untested assumption in the Introduction (Lines 559–561).

b. Although generally factually accurate, the introduction oversells the relationship of red blood cell mitochondrial function to tissue and whole animal metabolism. A careful look at the cited papers reveals that the correlation between RBC mitos and tissue mitos are either not very strong (< 0.7) or are more robust but with a very shallow slope that reveals very small changes in RBC mito respiration with larger changes in tissue respiration. The relationships are significantly correlated but the very low sensitivity of RBC assays relative to tissue assays suggests that the curve is very shallow and small, and thus has poor sensitivity to changes, which is fine for HRR approaches but likely contributes to very low resolution in the high throughput approach where tissues and assays are not as granular as with HRR. Similarly, the relationship between RBC respiration and whole animal metabolic rate correlated only with nighttime metabolism and not daytime metabolism, from which the cells were isolated…this suggests a bit of a contorted effort to squint and make a story work, rather than a robust correlation upon which to build the present study. I encourage the authors to present this introductory information in a more balanced fashion to reflect these less-than-ideal relationships as such.

We have revised the introduction (Lines xx-xx), not to oversell the relationship between RBC mitochondrial function and tissue/whole-organism metabolism, stating ‘However, it is important to note that these relationships are often modest in magnitude, and in some cases involve shallow slopes (i.e., comparatively small changes in RBC respiration corresponding to much larger changes in tissue or whole-organism metabolism), which likely limits the sensitivity of RBC-based assays to detect subtle biological effects. The present study uses avian blood cells primarily as a practical system for method comparison.’ (Lines 120-125). Avian RBCs have been used in the present study as a minimally invasive tissue for method comparison, as they demonstrated presence of functional mitochondria and sensitivity to experimentally imposed variation in temperature and species, not because of their capacity to serve as a proxy for whole-animal metabolism.

2. The authors ignore existing products that achieve many of their aims. Isn't the Agilent Seahorse basically a plate reader that measures oxygen consumption in real time and at relatively high throughputs than HRR? What is novel about the current approach in terms of the technique and design? I recognize that this approach doesn't require the purchase of a Seahorse but it does require the purchase of a plate reader, if one isn't already available. In either case, the current discussion of options available to researchers (HRR and the present approach) ignores this key existing technology. Please include discussion of where the seahorse fits into the field in the introduction to appropriately position the need for the present approach.

© 2026. Published by The Company of Biologists under the terms of the Creative Commons Attribution License (https://creativecommons.org/licenses/by/4.0/).

We agree that the Agilent Seahorse XF Analyzer represents a key existing technology that sits between HRR and a general-purpose plate reader, and that our original Introduction was remiss in not discussing it. We have now added a paragraph outlining the use of the Seahorse following the introduction of HRR and preceding our plate-reader approach (Lines 87–102). The novelty of our approach is not linked to the underlying measurement principle, fluorescence-based oxygen detection has been used before, but in its accessibility: standard fluorescence microplate readers are more common in biological laboratories and serve a wide range of assay types, making them available to research groups that do not already have access to dedicated mitochondrial respirometry instrumentation, such as an Oroboros O2k or Seahorse analyser, and for whom the cost of acquiring one would be difficult to justify given their broader research priorities. The Seahorse, while higher throughput than HRR, remains a dedicated and costly piece of equipment, and consumable costs per run are considerable. Our approach therefore fills a distinct niche for labs seeking a low-cost, low- barrier entry point into mitochondrial respirometry using equipment they are likely to already own or have more justification to purchase.

3. Methods: I am unclear regarding the workflow for blood collection and experimentation. The authors describe collecting blood and processing it within 2 hours but they also describe measuring the same blood with three different temperatures in assays that are each 90 minutes long on a single plate reader. Something doesn't add up here. Were samples used across the full ~ 5+ hours of time required to support these 3 different temperature runs on microplates? But then the authors say the assay was run across 3 days…so does this mean the same blood sample was kept on ice for 72 hours across these assays and tested each day? This would be concerning and evidence that the blood and mitochondria are still highly viable after 72 hours would be necessary. Overall, I found the description of tissue handling and workflow to be very hard to decipher.

We thank the reviewer for highlighting this confusion, which reflects ambiguity in our original description. For clarification, all three temperature measurements were performed on the same day using a single individual's blood sample collected that morning. Blood was collected from a subset of different individuals each morning, and all three temperature runs were completed on the same day, with the total handling time remaining within acceptable limits while stored at 4°C in Mir05 (i.e., <12 hours). Different individuals were measured on different days across the three-day experiment and blood was never stored overnight. We have rewritten the relevant passage in the Methods to make this same-day workflow clearer (Lines 175–177 and lines 200-203).

4. Duration: 90 minutes is quite a long assay time for mitochondrial measurements and may lead to degeneration of the quality of the preparation. The authors indicate that they took measurements throughout this period but it is not clear what period was used for the final analysis….was it averaged across the full 90 minutes? It would be useful to see the rates across time with each 90 sec bin plotted so that the reader can evaluate how assay time is impacting the respiration rate of mitochondria in this environment.

We did not average across the full 90 minutes and apologise for this confusion in the methods section. Rather, the respiration rate for each well was determined by calculating slopes over sliding windows of four consecutive time points (~6 minutes) and selecting the maximum slope within the linear region of the curve (see figure below). This approach captures the peak respiration rate before any potential deterioration and avoids the inclusion of later time points where signal quality may have declined. While this was specified in the original submission, we have clarified this further in the Methods section (Lines 287–299). Representative fluorescence curves across the full 90-minute assay period like the one provided below, with the selected analysis window indicated, will be provided as part of the supplementary R code and HTML outputs included in the data repository. This was referenced in the original submission as well but not reproduced in the manuscript itself. We have now explicitly directed readers to this resource in the Methods section (Lines 299–301).

© 2026. Published by The Company of Biologists under the terms of the Creative Commons Attribution License (https://creativecommons.org/licenses/by/4.0/).

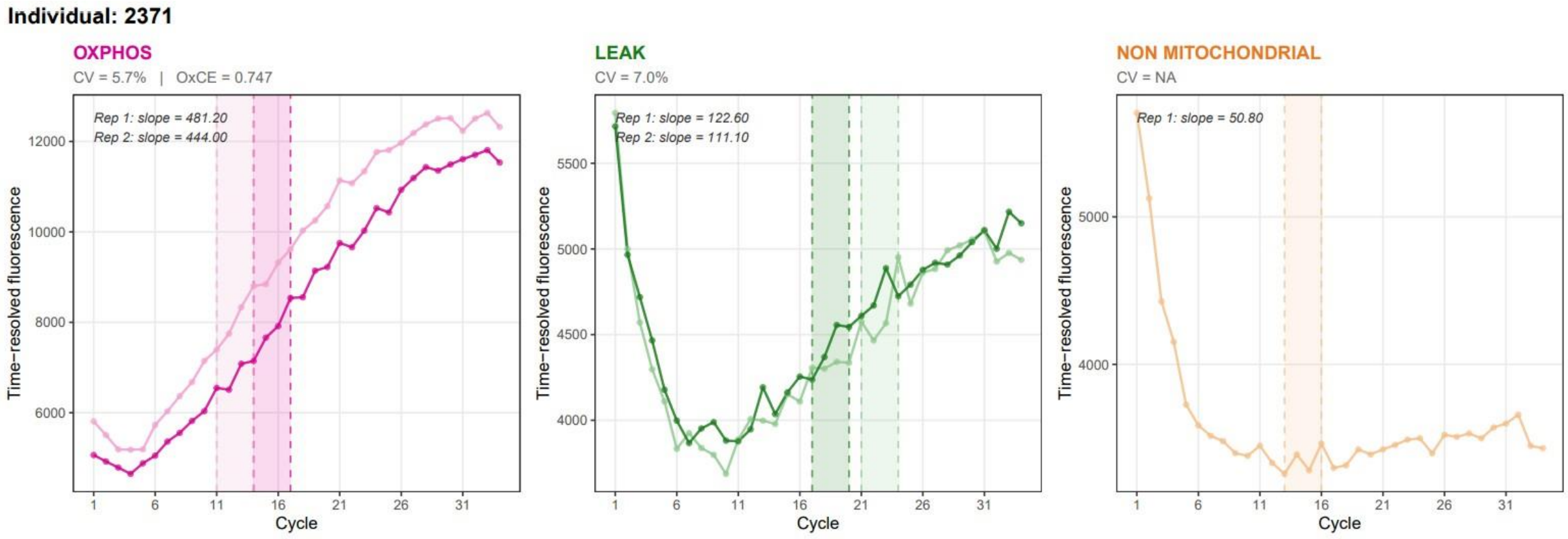

5. Mito quality: There is a clear and very significant difference in OxCE between preparations, both in terms of the large difference between means in all species and conditions but also the large variability apparent in the plate spec assay relative to the very tight datasets for the HRR. Many of the values for OxCE are quite low and the threshold of 0.4 likely includes a lot of mitochondria that are in rough shape. Usually, it is ideal to see numbers of > 0.8 for healthy and reliable mitochondrial preparations. I think the huge differences in mean and variability between preparations clearly demonstrate that the plate assay is not recapitulating the data obtained from HRR and call into question the viability of the plate prep.

Applying HRR-derived quality thresholds (e.g. OxCE > 0.8) to plate-based fluorescence measurements is not straightforward, as the two methods differ fundamentally in their measurement principles and cannot be expected to yield identical absolute values for ratio-based metrics such as OxCE. In response to this comment, we also revisited our OxCE calculation for the plate reader. Our original calculation did not subtract non-mitochondrial respiration from the plate-reader OXPHOS and LEAK values before computing OxCE; we have since corrected for this, in line with standard practice in the field and consistent with how OxCE is calculated for the HRR dataset (see Methods). This correction raised plate-reader OxCE values overall (and thus improved the correlation in Experiment 1 (Fig. 2). The field has not adopted a universal OxCE threshold for plate-based methods equivalent to the HRR standard of > 0.8; we therefore revised our empirical threshold accordingly, from 0.4 to 0.5, based on both the distribution of values in our updated dataset and published values for avian blood cells from other labs (e.g. in permeabilized avian blood cells; Ton et al., 2025: [0.50-0.88]; Correia et al., 2025: [0.50-0.92]).

Because OxCE is calculated as (OXPHOS−LEAK)/OXPHOS, its lower values on the plate reader cannot simply reflect a uniform reduction in both OXPHOS and LEAK, but rather a disproportionate difference between the two: OXPHOS correlated significantly with HRR in both experiments and the pooled analysis, whereas LEAK did so only in Experiment 1, pointing to LEAK being overestimated on the plate reader. If mitochondrial preparations were generally of poor quality, as the reviewer suggests, OXPHOS should be affected as well, since damaged mitochondria cannot properly couple ADP phosphorylation to respiration; its consistent agreement with HRR argues against this and instead points to a trait-specific issue with LEAK measurement. Notably, in our newly added second experiment, OxCE correlated significantly between the plate reader and HRR, further supporting that the Experiment 1 discrepancy reflects a condition-dependent measurement issue with LEAK rather than poor mitochondrial viability. We have revised the relevant Methods and Discussion sections accordingly (Lines 240–242 and Lines 326–333).

6. Temperature effects and sensitivity. The HRR data returned the expected relationship such that with increasing temperatures, respiration rate increases (and vice versa with colder temperatures). However, this is clearly not recapitulated in the plate reader data. There seems to be a total failure of the HSS assay to measure the impact of this experimental

© 2026. Published by The Company of Biologists under the terms of the Creative Commons Attribution License (https://creativecommons.org/licenses/by/4.0/).

condition on mito respiration in RBCs in either species. If the plate based assay cannot detect temperature- dependent changes (which, from the HRR data are robust and clear) then this calls into question the ability of this assay to reliably detect biological changes in a wide variety of testable conditions…or species differences that are not as robust as happened to emerge between the two birds studied herein.

We understand the reviewer's concern regarding whether the plate-based assay is able to detect true biological changes. We agree that the plate reader's inability to fully recapitulate the temperature- respiration relationship seen with HRR reflects a genuine limitation of the assay as currently implemented. As discussed in the manuscript, this appears to stem from two compounding factors. First, HRR's finer resolution allows more sensitive detection of variation at low fluxes, and this limitation was most apparent in our chicken data from Experiment 1, where lower respiration rates were associated with larger residual variance and wider confidence intervals for plate-reader estimates (Lines 596–601). Second, at higher temperatures (e.g., 44°C), the plate reader's slower setup and measurement initiation likely allowed blood cells to experience thermal stress before measurement began, whereas HRR measurements are taken immediately after substrate addition and better preserve cellular integrity under these conditions (Lines 601–605).

In line with this, we have revised our conclusions to state that the plate-based assay may be reliable for characterising variation under near-physiological temperatures and sufficiently high flux conditions, and that HRR remains better suited to exploring mitochondrial performance at the edges of the thermal or metabolic envelope, and that further testing is needed before extending the plate reader's use to conditions beyond near-physiological temperatures (Lines 605–608).

In support of this, our newly added second experiment, conducted only at 40°C, showed that the plate reader reached the same conclusions as HRR for LEAK (no significant sex effect detected by either method) and OxCE (significant with both methods), and yielded highly similar effect sizes for OXPHOS, with HRR reaching significance narrowly ($p = 0.046$) and the plate reader falling just short ($p = 0.060$). This is consistent with our broader conclusion that the plate reader performs most reliably, and with greater statistical power, under near-physiological temperatures and for traits with sufficiently high flux, even if its comparatively greater measurement noise can still shift borderline effects across the significance threshold.

7. Data variability. The data is highly variable and the ability to detect changes is suspect. The authors note this themselves when describing how estimates "differed in magnitude and direction between methods and species." That is not a reliable assay! The authors conclude that the plate reader assay can be accurate under "specific assay conditions" but these conditions are not known unless HRR is conducted in parallel to fact-check the data of the plate reader. In this case the temperature data was not useful but some of the species comparisons were…but in a novel experiment a researcher wouldn't know what data was real and what was not without conducting the more time-consuming assay. As such, the chances of false positives and false negatives is very very high, which is not useful for plate-based high throughput assays. The authors conclude that this assay may not be good for looking at the effects of temperature but how would one know if it is effective at looking at the effects of other treatments without the same control approaches? The fact that some differences were detectable is really more about luck/chance and I don't think reflects widespread reliability in this method. How is a researcher to know what is a "real" change and what is a failure of the system to detect a change? The error rate will be enormous and significant and constantly provide researchers with incorrect information. The authors suggest that with higher sample numbers the values would average out and become more accurate but as the paper stands there is really very little data to support their conclusions, and the bulk of data suggests that the plate based assay is not at all appropriate for these research questions. If the authors wish to proceed with this claim then at the very least they must show that it works. Additional experiments with higher replicates (384 well plates) to have sufficient sample sizes to reliably show the same relationships as HRR reveals is an absolute minimum requirement for publication of this data because otherwise there is no support for the authors present conclusions.

We agree that given the constraints of running both methods in parallel at scale, our

© 2026. Published by The Company of Biologists under the terms of the Creative Commons Attribution License (https://creativecommons.org/licenses/by/4.0/).

dataset does not necessarily have the replicate numbers needed to have a complete validated ready-to-use plate- based assay. We also do not wish to overstate what our data can support and have opted for caution, recommending future studies prioritise optimisation for their study systems.

However, we do not believe that the differences we detected reflect chance alone. As discussed in the manuscript (Lines 524-526 and 605-608), the plate reader performed well under specific and identifiable conditions (i.e., near-physiological temperatures, for higher-flux traits), while performance decreased under more challenging conditions (i.e., high temperature, low-flux traits). For example, LEAK is a comparatively low-flux trait at lower temperatures, and correlation between the plate reader and HRR for LEAK was correspondingly weak and non-significant at 36°C ($r = -0.07$, 95% CI: −0.64–0.55) and 40°C ($r = 0.32$, 95% CI: −0.31–0.76); however, as LEAK flux increases with temperature, this correlation became strong and significant at 44°C ($r = 0.74$, 95% CI: 0.37–0.91), consistent with the plate reader's performance improving once flux is high enough to be reliably resolved above measurement noise. Our newly added second experiment further supports this pattern: the plate reader agreed with HRR for LEAK and for OxCE, and yielded closely matching effect sizes for OXPHOS, with HRR reaching significance narrowly and the plate reader falling just short, consistent with condition-dependent reliability driven by measurement precision rather than random performance.

Instead of repeating the temperature comparison at higher replicate numbers (e.g., 384-well plates that would need another set of experimental conditions due to lower well volume and longer pipetting time before launching analysis), we chose to add a second, independent experiment testing the assay's ability to detect a different, source of biological variation (sex) under simpler, near- physiological conditions. We believe this more directly addresses the reviewer's core concern about the assay's reliability than would increasing sample size within the original, more challenging temperature- comparison design, since it tests whether the assays can agree on biological differences under conditions where we would expect the plate reader to perform best (i.e., normothermia, high flux).

In response to this and related comments, we have already revised our conclusions (Lines 709-723) to explicitly state that the plate-based assay's reliability is condition-dependent, and in what situations it might be best suited.

8. The cells spend a long time in the wells and may become hypoxic in that period. The high degree of variability and poor OxCE values suggest damaged and less functional mitochondria. The authors also suggest that cells at the higher temperature may have been exposed to heat stress before the assay began. Wouldn't this issue also spill over into other stress tests that researchers might potentially explore with this system? I would like to see some cell viability data for samples or other measures of mitochondrial viability (for cells treated IN the plate reader). Without quality controls, any mitochondrial results are relatively meaningless, and I fail to see any quality control built into the plate reader experiments at present, based on the criticism of the OxCE approach included in the Discussion section.

Mitochondrial quality control is essential for interpreting these data, and we would like to clarify how our study addresses this, while acknowledging where direct viability measures are missing.

Handling was identical between methods up to permeabilisation, as both HRR and plate reader assays used the same blood pool per individual, with digitonin from the same stock and concentration (amounts optimised separately per method). We therefore have no a priori reason to expect plate reader- derived cells to be less viable than those measured by HRR, particularly since OXPHOS correlates well with HRR throughout our dataset (see comment 5). Avian RBC mitochondrial respiration also remains measurable after 24 hours of cold storage, indicating these cells are robust over timescales well exceeding our pre-measurement window (Stier et al., 2016).

This robustness extends to the measurement window itself: although the full plate reader run lasts 90 minutes, we extract the maximum slope within the linear region, which occurs generally before ~30 minutes for OXPHOS and before ~40 minutes for LEAK. Thus, it is not at the 90-minute endpoint that a viability check would need to be targeted. Fluorescence shows an initial lag phase followed by a sustained linear rise, during which the maximum slope is extracted, with

© 2026. Published by The Company of Biologists under the terms of the Creative Commons Attribution License (https://creativecommons.org/licenses/by/4.0/).

no abrupt drop or irregular decline during or after this window, consistent with sustained mitochondrial activity and arguing against acute cell damage or catastrophic hypoxia (see figure provided in response to number 4, manuscript Fig. 1, and supplementary HTML document on our slope-extraction method). The sliding-window approach is designed to isolate this stable linear phase from the surrounding lag and any later flattening of the signal, rather than assuming uniform linearity across the full run. Further, if hypoxia were limiting, OXPHOS (the faster O2 consumer) would likely be affected first; instead, it correlates consistently with HRR, arguing against hypoxia as a meaningful constraint. See also our responses to comments 3 and 4 for further discussion of assay duration; we regret this was not clear in the original Methods.

Regarding the reviewer's point that poor OxCE values suggest damaged mitochondria: as detailed in our response to comment 5, this reflects LEAK being overestimated on the plate reader, not a general loss of viability. This is supported by our new second experiment, where OxCE correlated significantly with HRR. We agree, however, that we lack a direct, independent measure of viability or mitochondrial integrity (e.g., live/dead staining, cytochrome c addition) run in parallel on the plate reader and acknowledge this as a limitation; we have added a recommendation for future studies to include such controls (Discussion, Lines 655–660).

References

Stier et al. (2016) *Methods in Ecology and Evolution*. DOI: 10.1111/2041-210X.12724

9. Overstating the data. The authors conclude that the plate assay captured broadly similar patterns to HRR. The data does not support this. There is far greater variability and no ability to detect temperature based changes accurately between species. The assay does detect broad patterns between species when temperature is not compared but these differences are generally pretty large, likely reflecting the very low power of the plate based assays….and a lot of chance that these species happened to have large enough differences to measure with this very crude approach.

We agree that our original phrasing overstated the degree of agreement between the two methods, and we have revised the Discussion accordingly (Lines 524-526) to lead with a more accurate, condition-dependent framing rather than asserting that the plate reader broadly captured similar patterns to HRR across the board.

We take the reviewer's concern to be less about chance and more about effect size: the quail– chicken comparison happens to involve a very large mitochondrial difference, and a more modest between-species difference might not have been resolved by the plate reader even where HRR could detect it. This is a fair point given the assay's comparatively low power. To address it directly, we point to our newly added second experiment, where the plate reader reliably detected a considerably more subtle source of biological variation (i.e., a sex difference under normothermic conditions): it matched HRR's conclusions for LEAK (no significant effect detected by either method) and for OxCE (significant with both methods), and yielded an effect size for OXPHOS nearly identical to that of HRR (β = -0.84 versus -0.74), with HRR reaching significance narrowly ($p = 0.046$) and the plate reader falling just short ($p = 0.060$). This indicates that the assay's sensitivity is not limited to unusually large, species-level effects, and that where it departs from HRR, it does so as a marginal loss of statistical power on a borderline effect, rather than as a spurious, chance-driven signal (i.e., directly addressing the reviewer's concern about the assay generating unreliable, false-positive results). We fully acknowledge, however, that the plate reader's ability to resolve temperature effects specifically remains limited, as now stated explicitly in the revised Discussion.

Reviewer 2: Overview

'High-throughput mitochondrial aerobic metabolism measurement: validating a plate-reader approach using avian blood cells ' is a well-written and well-structured manuscript.

© 2026. Published by The Company of Biologists under the terms of the Creative Commons Attribution License (https://creativecommons.org/licenses/by/4.0/).

I appreciate the authors' effort in developing and validating a fluorescence-based microplate reader approach to assess mitochondrial aerobic metabolism, including oxidative phosphorylation (OXPHOS), leak respiration, and oxidative control efficiency (OxCE; the difference between OXPHOS and leak respiration), using blood samples from chickens and Japanese quail at three temperatures (36°C, 40°C, and 44°C). These temperatures represent hypothermic, normothermic, and hyperthermic conditions, respectively, in the study organisms. The authors demonstrate that, under normothermic conditions, measurements of OXPHOS and leak respiration obtained using the microplate reader assay correlate well with those obtained using the widely accepted high-resolution respirometry (HRR) approach with the Oxygraph O2k. However, these correlations appear weaker at hypothermic and hyperthermic temperatures. In addition, oxidative control efficiency did not show a significant correlation between the two methods at any of the tested temperatures. The development of a high-throughput method for estimating mitochondrial aerobic metabolism would represent a valuable advancement for the field. Such an approach could serve as an efficient screening tool prior to the application of more precise and labor-intensive HRR measurements. Therefore, this study has the potential to make a meaningful contribution to the mitochondrial physiology research community. Overall, the manuscript is comprehensive and thoughtfully discusses both the strengths and limitations of the proposed methodology.

We thank the reviewer for their thoughtful and encouraging assessment of our manuscript. We appreciate the recognition of both the potential value of a high-throughput plate-reader approach for estimating mitochondrial aerobic metabolism and our efforts to discuss its strengths and limitations relative to HRR. In response to the reviewer's specific comments, we have clarified the scope and constraints of the method, and we more clearly outline how this assay can be used as an efficient screening tool and as a basis for future species-specific validation and comparative work.

Reviewer 2: Specific Comments

---

10 (1) Lines 234-254 (Microplate-based fluorescent assay): As this is primarily a methods-focused manuscript, I recommend including the exact concentrations of all reagents used in the assay within the main text. The authors refer readers to the supplementary material for these details; however, I was unable to locate the complete reagent concentrations. Providing this information directly in the manuscript would improve reproducibility and accessibility.

All reagent concentrations used to obtain mitochondrial measurements are now specified in the main text (Lines 221-229), in addition to Table 1 (which presents the titration between methods) and to Table S1 and S2 in the supplementary materials (which details every product we used, their provider, and their CAS Number to facilitate reproducibility). We also added a sentence (Lines 153-158 and lines 236-237) to clarify that all concentrations mentioned in the above text are the same all along the methods.

11 (2) Lines 209-212: The authors used antimycin A to assess non-mitochondrial respiration. However, inhibitors such as potassium cyanide (KCN) or sodium azide are more commonly used for this purpose. I suggest that the authors provide a justification for their choice of antimycin A as the sole inhibitor and discuss any potential implications of this methodological decision.

Antimycin A blocks electron flow at complex III, which is downstream of both complex I and II, so it fully blocks mitochondrial respiration from both entry points, making it suitable for defining residual non-mitochondrial oxygen consumption. In this context, antimycin A is widely used to determine residual (non-mitochondrial) oxygen consumption in intact and permeabilised cells (Brand and Nicholls, 2011; Stier et al., 2017; Nord et al., 2021; Pesta & Gnaiger 2012). Although KCN and sodium azide are sometimes used for this purpose, these Complex IV inhibitors are not selective for the mitochondrial electron transport chain: they are known to inhibit haem-

© 2026. Published by The Company of Biologists under the terms of the Creative Commons Attribution License (https://creativecommons.org/licenses/by/4.0/).

containing non-mitochondrial enzymes, including NADPH oxidases and cytochrome P450s, which would artificially suppress the signal being measured and confound the quantification of non-mitochondrial respiration. Brand and Nicholls (2011) caution against the use of cyanide-based inhibitors for this reason, recommending antimycin A (± rotenone) as the preferred specific inhibitors for this application. We now explicitly state this rationale in the Methods section (Lines 229 – 236).

References

Brand, Nicholls (2011) *Biochemical Journal*. DOI: 10.1042/BJ20110162

Stier (2017) *Methods in Ecology and Evolution*. DOI: 10.1111/2041-210X.12724

Nord (2021) *The FASEB Journal*. DOI: 10.1096/fj.202100107R

Pesta, Gnaiger (2012) *Methods in Molecular Biology*. DOI: 10.1007/978-1-61779-382-0_3

12 (3) Figure 2: I recommend including separate regression lines for each temperature treatment within each parameter panel (OXPHOS, leak respiration, and OxCE). Additionally, presenting the correlations separately for chickens and Japanese quail would improve transparency and provide a clearer assessment of assay performance across species and temperature conditions.

Separate regression lines for each respiratory trait, measured across all three temperatures, have now been added on Fig. 2. Pearson coefficients and their respective confidence intervals are presented in Table S4 of the Supplementary materials. Concerning the addition of specific correlation per species and temperature, we decided to put them in the supplementary material as well, so as not to overload the main text of the manuscript (Fig. S3, Table S4).

13 (4). Discussion (Lines 473-475): The relationship between the microplate reader assay and HRR measurements may vary among species due to differences in blood cell physiology, mitochondrial characteristics, or thermal sensitivity. I encourage the authors to acknowledge this possibility and include species-specific validation as an important consideration for future studies.

We agree that the relationship between plate-reader and HRR measurements could differ among species due to variation in blood cell physiology, mitochondrial characteristics, and thermal sensitivity. This is an important limitation of any method validated in a single species. We have expanded our discussion of this point in the Discussion (Perspectives in Ecology and Evolution, Lines 679–687), noting that as this protocol continues to be developed and applied across taxa, a broader species comparison against HRR would be a valuable next step to better characterise how consistently the two methods align.

Second decision letter

MS ID#: bio.062712R1

MS Title: High-throughput mitochondrial aerobic metabolism measurement: assessing a plate-reader approach using avian blood cells

Authors: Cloé Hadjadji; Molly Ohse; Antoine Stier; Pierre Bize

© 2026. Published by The Company of Biologists under the terms of the Creative Commons Attribution License (https://creativecommons.org/licenses/by/4.0/).

This is a first for me: receiving two polar-opposite reviews. But, after careful reading of reviewer 2's comments, they are mainly concerned about the technique not being adequate enough to replace known techniques (especially the old-school electrode approach). I will give the authors the opportunity to respond to the concerns and will make a decision on whether I am satisfied with the responses.

At this stage, we also ask you to ensure your manuscript complies with our formatting guidelines. Provided you are able to fully address the referees' comments, we are positive about publication of your paper (we accept over 95% of revision submissions) and therefore hope you won't mind any extra work involved in reformatting your manuscript at this point.

Please upload both a 'clean' version of your Word file, along with a highlighted version clearly showing where you have made changes in the revised manuscript. Please avoid using 'Track changes' in Word files as these are lost in PDF conversion.

I should be grateful if you would also provide a point-by-point response detailing how you have dealt with the points raised by the reviewers in the 'Response to Reviewers' box. Please attend to all of the reviewers' comments. If you do not agree with any of their criticisms or suggestions please explain clearly why this is so.

Reviewer 1

Comments for the author

Overview: The authors have made a strong effort to address my concerns. I appreciate the inclusion of an additional experiment and the work that went into generating this data. However, the revisions and comments of the authors do not address my fundamental concerns with this study. Specifically, although the assay offers a high throughput means to examine mitochondrial function in avian red blood cells under certain conditions, the signal to noise ratio remains very large, the assay lacks a validated and robust quality control mechanism, and the assay seems to only be useful in one cell type of birds, and then only if measured in normothermic conditions, and then still produces noisy data with low power to detect changes. Overall, I struggle to see the utility of this assay beyond this narrow application and the fit for such a specific assay seems awkward in the present journal. I offer the following comments to support my conclusion.

Major concerns:

1. Quality control: the authors use OxCE as the sole measure of mito viability but also state that this measure on a plate reader can't correspond to acceptable values in the HRR. But, then then they allow themselves a much larger range of "acceptable" values based on studies conducted in HRR. However, they also state that in their initial experiments, samples exposed to non-normothermic temperatures may have been damaged by the high temperature of the plate reader resulting in more variability and noise in their data (they don't explain why OxCE values were also quite bad for the lower temperature, which should not have been damagaing, but which may have been do to a lower respiration rate reducing the signal/noise ratio on the numerator side of that equation). BUT none of those samples exceeded their chosen cut-off of 0.5 OxCE and are thrown out. This is not ideal. How can data be described as likely skewed by unhealthy mitos but still meet the threshold for healthy mitos and not be put aside?

If OxCE is to be used as the only measure of mitochondrial quality in this plate assay, then it must be calibrated for THIS assay and stricter cut-offs likely need to be applied if lower OxCE does indeed correspond to damaged mitos. Specifically, the authors must use other assays to determine if mitos are viable at various time points and treatments to confirm that this lower OxCE threshold is appropriate. It is important to conduct these control experiments in the plate reader itself as assays conducted on cells from a stock may not reflect the impact of storing cells in a plate reader without oxygenation and at higher tempratures for the experimental duration. RCRs or changes with cytochrome c addition are standard options, as are cell death/viability assays that are optimized for plate readers…of which there are many. As the assay currently stands, I am not

© 2026. Published by The Company of Biologists under the terms of the Creative Commons Attribution License (https://creativecommons.org/licenses/by/4.0/).

confident in the quality control measures, particularly as the authors themselves point to poor OxCE values from experiments that are both above and below the normothermic values as being likely skewed by unhealthy mitos. For a high throughput assay to work, researchers must be very confident in the criteria to exclude damaged samples and that has not been developed in this paper.

2. Signal to noise ratio: the assay remains very noisy and with a low power to reliably detect biological changes. I appreciate that the authors show somewhat similar results between the two approaches in their new experiment, but this highlights my concern. The first experiment provided very poor correlation between the two approaches, the second experiment was better but still not great. The authors point to the difference being due to a lot of noise, a low power signal, and possibly damaged mitos, but have no means to address any of these limitations. In a high throughput assay. One must be very confident in the results of a single readout to look at a large number of potential interactions and treatments. In my opinion, the present assay is not sufficiently robust to reliably detect these changes in experiments without further validation with HRR to confirm the results.

3. Assay utility. I appreciate the work the authors have done to show that their assay works a bit better in a comparison across animals in normothermia, but this also highlights the limited utility of this assay. Overall, it seems the assay is useful in RBCs that can sit out for 12+ hours on ice, and RBCs have only a moderate correlation with other tissues to mitochondrial function. It is potentially useful in birds, but then only in normothermia. Furthermore, because the quality control readout is minimal and not validated for this assay, other treatments beyond temperature that impact mitochondrial viability and health may skew the results similarly to assay temperature. Thus, this seems like an assay that has a very narrow utility and is likely not adaptable to studies in species that have lower metabolic rates (and thus likely even lower signal to noise ratios)…inverts, fish, some mammals, amphibians, reptiles…none of these could be studied using this assay. Given this, the general biology nature of the present journal seems like a poor fit for this study. I think that to be published in a general biology journal, the assay needs to be fully developed and some evidence should be provided that it works in tissues/species beyond bird RBCs only.

4. I am concerned by the approach of taking the steepest slope in 6 min bins to assess mitochondrial respiration as it appears their approach might lead to bias. For example, sample plots provided by the authors show how a 6 minute bin can look like a fairly big increase in respiration rate on what is really a flat relationship. Specifically, the non-mitochondrial panel in the response to reviewers shows quite a steep increase in the 6-minute bin but the overall slope across cycles 11-30 is pretty flat.

5. I appreciate the authors including information about the seahorse but they are still omitting the basic clark-type electrode means of measuring mito function. The oroboros is basically a very fancy version of this but the old-school electrode approach to measuring O2 consumption from mitos is very cost-effective and requires minimal specialized equipment. I would argue this approach is cheaper than the plate-spec assay. Thus, the major advantage of the present assay is speed of throughput and NOT cost or availability of equipment.

Reviewer 2

Comments for the author

The revised version of the manuscript, "High-throughput mitochondrial aerobic metabolism measurement: validating a plate-reader approach using avian blood cells" , meets all the necessary changes asked by the reviwers. I appreciate the authors' effort and time for considering the suggestions and making the modifications.

To my understanding this manuscript will add a valueable piece in the field of mitochondrial physiology and bioenergetics. I enjoyed reading it and am looking forward for it to be published.

Reviewer's Responses to Questions

© 2026. Published by The Company of Biologists under the terms of the Creative Commons Attribution License (https://creativecommons.org/licenses/by/4.0/).

Experimental quality
Are experiments performed using appropriate methods that will answer the question (or test the hypothesis or support the observations) posed by the authors? Is the right tool used for the job?
If 'No', please indicate reasons in Comments for Author box below.
Reviewer #1:
- No

Reviewer #2:
- Yes

Were the data analyzed using appropriate statistical tests?
If 'No', please indicate reasons in Comments for Author box below.
Reviewer #1:
- Yes

Reviewer #2:
- Yes

Reproducibility
Were experiments performed using adequate number of biological replicates?
If 'No', please indicate reasons in Comments for Author box below.
Reviewer #1:
- No

Reviewer #2:
- Yes

Is there sufficient raw data to assess the rigor of the analysis?
If 'No', please indicate reasons in Comments for Author box below.
Reviewer #1:
- No

Reviewer #2:
- Yes

Does the methods section provide sufficient detail to permit reproducibility?
If 'No', please indicate reasons in Comments for Author box below.
Reviewer #1:
- Yes

Reviewer #2:
- Yes

Completeness
Are the manuscript's conclusions supported by the data?
If 'No', please indicate reasons in Comments for Author box below.
Reviewer #1:
- No

Reviewer #2:
- Yes

© 2026. Published by The Company of Biologists under the terms of the Creative Commons Attribution License (https://creativecommons.org/licenses/by/4.0/).

Scholarship
Do the authors cite and discuss the merits of data that would argue for and against their conclusion?
If 'No', please indicate reasons in Comments for Author box below.
Reviewer #1:
- Yes

Reviewer #2:
- Yes

Does the manuscript title & abstract accurately reflect the contents of the manuscript, without hyperbole?
If 'No', please indicate reasons in Comments for Author box below.
Reviewer #1:
- Yes

Reviewer #2:
- Yes

Second revision

Author response to reviewers' comments

Dear Editor,

We are pleased to submit the second-round revisions of our paper entitled "High-throughput mitochondrial aerobic metabolism measurement: assessing a plate-reader approach using avian blood cells" which we would like to see published in *Biology Open*. Please find hereafter the comments from the reviewers and our responses. In bold are the comments of the reviewers and in normal font are our responses.

Overall, Reviewer 2 raised no further concerns, and confirmed that our previous revisions addressed their comments in full. Reviewer 1, however, raised additional concerns, that we addressed as follows:

- Ø We added a new validation experiment using cytochrome *c* to directly test outer mitochondrial membrane integrity under our plate-reader protocol; the results, now included in the manuscript (Methods; Fig. S4), which shows no sign of mitochondrial disruption at normothermia (40°C), whereas there was little evidence of mitochondrial disruption at hypothermia (36°C; 1/12 of the samples), but relatively marked evidence at hyperthermia (44°C; 4/12 of the samples). It supports the use of mitochondrial respiration using the plate-reader approach at normothermia (our previous conclusion), and that lower OxCE on the plate reader compared to the HRR Oroboros is not attributable to loss of membrane integrity.
- Ø We have added information about Clark-type oxygen electrodes to the Introduction and Discussion, addressing this established, cost-effective method and clarifying how it relates to the throughput and cost trade-offs of our plate-reader approach.
- Ø We have clarified in the manuscript that the plate-reader assay is best understood as a high-
throughput complement to established respirometry methods under specific, validated conditions, rather than as a general-purpose replacement for them.
- Ø We have also revised our discussion of quality control and data extraction more broadly in response to the reviewers' remaining technical concerns.

We thank Reviewer 1 again for their comments and suggestions, which we have considered carefully (see below for detailed responses). We believe that the additional changes made to this second

© 2026. Published by The Company of Biologists under the terms of the Creative Commons Attribution License (https://creativecommons.org/licenses/by/4.0/).

revision of our manuscript have further improved our manuscript, and we hope that, together with our responses, it is now acceptable for publication in *Biology Open*.
Thank you very much for considering our work and revisions.
Kind regards,

On behalf of co-authors of the revised version of the manuscript **bio.062712**, Cloé Hadjadji

Reviewer 1:

Overview: The authors have made a strong effort to address my concerns. I appreciate the inclusion of an additional experiment and the work that went into generating this data. However, the revisions and comments of the authors do not address my fundamental concerns with this study. Specifically, although the assay offers a high throughput means to examine mitochondrial function in avian red blood cells under certain conditions, the signal to noise ratio remains very large, the assay lacks a validated and robust quality control mechanism, and the assay seems to only be useful in one cell type of birds, and then only if measured in normothermic conditions, and then still produces noisy data with low power to detect changes. Overall, I struggle to see the utility of this assay beyond this narrow application and the fit for such a specific assay seems awkward in the present journal. I offer the following comments to support my conclusion.

We thank the reviewer for their continued careful engagement with this manuscript. Below, we address each concern in detail, but would like to summarise our position here. We do not believe this assay needs to replace HRR, the Seahorse, or Clark-type electrodes to be a useful contribution: we present it, and have revised the manuscript throughout to state explicitly, as a high-throughput complement to these existing methods for specific, well-defined applications (i.e., near-normothermic conditions and higher-flux respiratory traits) rather than as a general-purpose substitute. We would note that normothermia is the condition under which the large majority of mitochondrial respirometry studies are actually conducted, which is precisely why we designed our second experiment around it: this is not a narrow edge case but the setting in which this assay would typically be applied. We also state in the Discussion that “the plate-reader approach described here should be applicable, after appropriate optimisation, to other tissues and species with sufficiently high O2 fluxes.” (Lines 767 – 768). Within that scope, we believe our data support the assay's usefulness, as demonstrated for example by Experiment 2, where similar meaningful differences between sexes were detected using the plate reader and HRR approaches. Finally, we added a new validation experiment using cytochrome c to directly test outer mitochondrial membrane integrity under our plate-reader protocol. It provides no evidence of disruption of mitochondrial respiration at 40°C.

Major concerns:

1. Quality control: the authors use OxCE as the sole measure of mito viability but also state that this measure on a plate reader can't correspond to acceptable values in the HRR. But, then then they allow themselves a much larger range of "acceptable" values based on studies conducted in HRR. However, they also state that in their initial experiments, samples exposed to non-normothermic temperatures may have been damaged by the high temperature of the plate reader resulting in more variability and noise in their data (they don't explain why OxCE values were also quite bad for the lower temperature, which should not have been damagaing, but which may have been do to a lower respiration rate reducing the signal/noise ratio on the numerator side of that equation). BUT none of those samples exceeded their chosen cut-off of 0.5 OxCE and are thrown out. This is not ideal. How can data be described as likely skewed by unhealthy mitos but still meet the threshold for healthy mitos and not be put aside? If OxCE is to be used as the only measure of mitochondrial quality in this plate assay, then it must be calibrated for THIS assay and stricter cut-offs likely need to be applied if lower OxCE does indeed correspond to damaged mitos. Specifically, the authors must use other assays to determine if mitos are viable at various time points and treatments to confirm that this lower OxCE threshold is appropriate. It is important to conduct these control experiments in the plate

© 2026. Published by The Company of Biologists under the terms of the Creative Commons Attribution License (https://creativecommons.org/licenses/by/4.0/).

reader itself as assays conducted on cells from a stock may not reflect the impact of storing cells in a plate reader without oxygenation and at higher tempratures for the experimental duration. RCRs or changes with cytochrome c addition are standard options, as are cell death/viability assays that are optimized for plate readers…of which there are many. As the assay currently stands, I am not confident in the quality control measures, particularly as the authors themselves point to poor OxCE values from experiments that are both above and below the normothermic values as being likely skewed by unhealthy mitos. For a high throughput assay to work, researchers must be very confident in the criteria to exclude damaged samples and that has not been developed in this paper.

We would like to clarify why low OxCE at both temperature extremes does not, in our view, point to a single explanation of damaged mitochondria, and why our 0.50 cutoff was not intended to function as a fine-grained viability diagnostic.

At 36°C, we believe the more likely explanation is the one the reviewer's own comment suggests: OxCE is a ratio, and at lower assay temperatures both OXPHOS and LEAK fluxes are lower in absolute terms, which reduces the signal-to-noise ratio on both terms of the ratio and inflates its variance without necessarily reflecting reduced mitochondrial function. This is distinct from the 44°C case, where we propose that brief thermal stress during plate sealing and loading (cells are exposed to the pre-warmed, 44°C mineral oil for approximately 30 seconds to one minute before the reader begins recording) may initiate mitochondrial stress that continues to compound over the subsequent 90-minute measurement window, since cells remain at this elevated temperature throughout the recorded run rather than only during the initial loading period. We have updated the manuscript accordingly to reflect this combined mechanism (Discussion, Lines 656–668).

If general mitochondrial damage were driving the pattern at either temperature, we would expect OXPHOS (higher-flux, more robust of the two traits) to be similarly compromised; instead, OXPHOS correlates consistently and significantly with HRR across Experiment 1, Experiment 2, and the pooled dataset, which argues against a general loss of viability and points instead to LEAK-specific measurement imprecision.

We would also clarify the intended function of our 0.50 threshold, and directly address why it excluded no samples in either experiment (plate-reader OxCE range: 0.51–0.98). This is separate from the exclusion criteria described in our data-cleaning section, which did remove a subset of measurements prior to analysis on other grounds (weak HRR signal linked to residual inhibitors; biologically implausible negative LEAK slopes). The 0.50 cutoff was set as a coarse floor to exclude clearly non-functional preparations, not as a diagnostic capable of distinguishing subtler reductions in performance, such as added noise or mild uncoupling, from fully intact preparations; values near this floor, including several of ours at 44°C, may therefore still reflect reduced signal quality without falling into a range we would consider non-functional. We agree a threshold serving that stronger, more sensitive purpose would require calibration against an independent, graded measure of mitochondrial function; our cytochrome c experiment provides independent evidence of outer-membrane integrity specifically, but does not itself calibrate the OxCE threshold, which reflects coupling efficiency rather than membrane integrity, and we already state this explicitly as a limitation (Discussion, Lines 713–716).

On the specific quality-control approaches the reviewer suggests: we would note that RCR and OxCE are not independent measures. In permeabilised-cell preparations such as ours, RCR is conventionally defined as OXPHOS/LEAK (Chance & Williams, 1955), and our OxCE, (OXPHOS−LEAK)/OXPHOS, is a direct transformation of the same quantity (OxCE = 1 − 1/RCR; Gnaiger, 2020). Thus computing RCR from the same OXPHOS and LEAK values would not provide new or independent information beyond what OxCE already captures.

We agree, however, that cytochrome c addition would offer genuinely independent information, since it tests outer mitochondrial membrane integrity directly rather than coupling efficiency. We have therefore conducted a validation experiment to test this directly within the plate reader: in 12 Japanese quail not included in Experiment 1 or 2, duplicate $OXPHOS_{CI+CII}$ wells with cytochrome c added to the buffer (10 μM final concentration) were compared against

© 2026. Published by The Company of Biologists under the terms of the Creative Commons Attribution License (https://creativecommons.org/licenses/by/4.0/).

duplicate OXPHOS wells without cytochrome c, prepared and loaded identically, at each of the three assay temperatures. Cytochrome c testing was restricted to OXPHOS, following established guidance that cytochrome c effects should not be assessed in the LEAK state, where respiration can increase for reasons unrelated to outer membrane injury (Oroboros Instruments, Bioblast: "Cytochrome c control efficiency"). Because cytochrome c cannot cross an intact outer mitochondrial membrane, a meaningful increase in OXPHOS respiration upon its addition indicates membrane damage, whereas minimal change supports membrane integrity; following convention (Kuznetsov et al., 2008), we considered a percent difference exceeding 10% indicative of a compromised outer membrane. As now reported in our Discussion section, this validation experiment "assessing mitochondrial integrity under the plate-reader protocol by adding cytochrome c to OXPHOS respiration showed a higher proportion of samples exceeding the integrity threshold at 44°C than at 36°C or 40°C (see Results), offering a plausible additional explanation for the reduced agreement between methods at 44°C, alongside the lower sensitivity of the plate reader noted above. Taken together, these results are broadly consistent with the plate-reader approach performing most reliably under stable, normothermic conditions." (Lines 583-584). We also have added the full description of the methods and results of this validation experiment to the manuscript (Methods, Lines 312-328 and 422-425; Results, Lines: 551-560; Fig. S4).

We did not pursue cell-viability assays as an additional independent check, however, as these assays report on plasma membrane integrity, and permeabilisation (the step that allows exogenous substrates to reach the mitochondria in our assay) necessarily compromises the plasma membrane. Cell- viability assays would therefore be expected to classify all of our (permeabilised) samples as non-viable regardless of their actual mitochondrial function, and so cannot serve as a meaningful quality-control measure for this preparation.

References

Chance, B., & Williams, G. R. (1955). *Journal of Biological Chemistry.*

Gnaiger, E. (2020). *Bioenergetics Communications* https://doi.org/10.26124/bec:2020-0002

Kuznetsov (2008). *Nature Protocols* https://doi.org/10.1038/nprot.2008.61

Oroboros Instruments. Cytochrome c control efficiency. Bioblast.
https://wiki.oroboros.at/index.php/Cytochrome_c_control_efficiency

2. Signal to noise ratio: the assay remains very noisy and with a low power to reliably detect biological changes. I appreciate that the authors show somewhat similar results between the two approaches in their new experiment, but this highlights my concern. The first experiment provided very poor correlation between the two approaches, the second experiment was better but still not great. The authors point to the difference being due to a lot of noise, a low power signal, and possibly damaged mitos, but have no means to address any of these limitations. In a high throughput assay. One must be very confident in the results of a single readout to look at a large number of potential interactions and treatments. In my opinion, the present assay is not sufficiently robust to reliably detect these changes in experiments without further validation with HRR to confirm the results.

We would like to clarify the strength of the correlations we report, as we believe "poor" and "not great" understate them. Pearson's r for OXPHOS and OxCE ranged from 0.42–0.60 across Experiment 1, Experiment 2, and the pooled normothermic dataset which are considered moderate-to-large effect sizes by conventional benchmarks (Cohen, 1988; Brydges 2019), and comparable in magnitude to cross-platform agreement reported for other high-throughput respirometry systems validated against chamber-based methods (e.g. Seahorse vs. Oroboros in human fibroblasts; Zdrazilova et al., 2022). LEAK was the one trait that did not correlate reliably outside Experiment 1, and we agree this is a genuine limitation, which we state plainly throughout the manuscript (Lines 681-683).

© 2026. Published by The Company of Biologists under the terms of the Creative Commons Attribution License (https://creativecommons.org/licenses/by/4.0/).

We also do not think we have "no means to address" these limitations. Our Discussion already sets out concrete paths to reduce measurement error (or noise) in future applications: increasing per- well signal magnitude (e.g. higher cell loading) (Lines 721-723), developing an absolute oxygen calibration (Lines 699-701), and adopting an intracellular probe to access additional respiratory states (Lines 710-713).

Finally, we would push back on the expectation that a high-throughput screening tool should not require downstream confirmation and that one must be very confident in the results of a single readout. This is standard practice for essentially every high-throughput method used as a first pass, screening approaches trade some individual-measurement certainty for the ability to survey many samples or conditions, with confirmatory follow-up reserved for effects of interest. We have consistently framed the plate reader this way throughout the manuscript, from the original submission onward: as a complement to HRR for identifying candidate effects under near-normothermic, higher- flux conditions, not a stand-alone replacement.

References

Brydges (2019). *Innovation in Aging*. https://doi.org/10.1093/geroni/igz036

Cohen (1988). *Statistical Power Analysis for the Behavioral Sciences*. https://doi.org/10.4324/9780203771587

Zdrazilova et al. (2022). *PLoS ONE* https://doi.org/10.1371/journal.pone.0264496

3. Assay utility. I appreciate the work the authors have done to show that their assay works a bit better in a comparison across animals in normothermia, but this also highlights the limited utility of this assay. Overall, it seems the assay is useful in RBCs that can sit out for 12+ hours on ice, and RBCs have only a moderate correlation with other tissues to mitochondrial function. It is potentially useful in birds, but then only in normothermia. Furthermore, because the quality control readout is minimal and not validated for this assay, other treatments beyond temperature that impact mitochondrial viability and health may skew the results similarly to assay temperature. Thus, this seems like an assay that has a very narrow utility and is likely not adaptable to studies in species that have lower metabolic rates (and thus likely even lower signal to noise ratios)…inverts, fish, some mammals, amphibians, reptiles…none of these could be studied using this assay. Given this, the general biology nature of the present journal seems like a poor fit for this study. I think that to be published in a general biology journal, the assay needs to be fully developed and some evidence should be provided that it works in tissues/species beyond bird RBCs only.

Mitochondrial aerobic metabolism is a core topic in cellular and organismal physiology, which is explicitly within Biology Open's scope and readership. The journal's stated scope spans the breadth of the biological and biomedical sciences, including physiologists working in ecological and evolutionary contexts such as ours.

We would push back specifically on the claim that full validation across species and tissues is a precondition for publication in a general biology journal: a journal's breadth of scope describes the range of topics and systems it publishes across collectively, not a requirement that any single study demonstrate universal applicability on its own. Single-species, single-tissue has been so far the standard trajectory for new respirometry methods: the original HRR protocol for avian blood cells that underlies the wider body of avian mitochondrial ecophysiology work we cite throughout this manuscript was itself first established and validated in a single system using RBCs (Stier et al., 2017) before being adopted broadly, including in other taxa such as fish (Jego et al. 2024). The same trajectory traces back to the foundational polarographic method itself, first validated exclusively in isolated rat liver mitochondria (Chance & Williams, 1955) before becoming, over subsequent decades, the basis for respirometry across essentially every animal system studied today. In other words, single-system validation followed by broader field-wide extension is not a peculiarity of avian mitochondrial ecophysiology, it is rather how respirometry as a discipline has always progressed, from its mammalian origins to now. Establishing feasibility and reliability in one

© 2026. Published by The Company of Biologists under the terms of the Creative Commons Attribution License (https://creativecommons.org/licenses/by/4.0/).

well-characterised system, with extension to further taxa flagged explicitly as a next step (Perspectives, Lines 742-745 and 767-768), is the expected first stage for any new method, not a shortcoming of this one.

In conclusion, the aim of our study is not to validate the applicability of the plate-based approach across every tissue and species, but rather to demonstrate its potential and to clearly define its current boundaries. We do not believe this places the study outside the journal's scope, but we leave that determination to the editor's judgement.

References

Jego et al. (2024). *Ecological and evolutionary physiology*. https://doi.org/10.1086/734065

Stier et al. (2017). *Methods in Ecology and Evolution*. https://doi.org/10.1111/2041-210X.12724

4. I am concerned by the approach of taking the steepest slope in 6 min bins to assess mitochondrial respiration as it appears their approach might lead to bias. For example, sample plots provided by the authors show how a 6 minute bin can look like a fairly big increase in respiration rate on what is really a flat relationship. Specifically, the non-mitochondrial panel in the response to reviewers shows quite a steep increase in the 6-minute bin but the overall slope across cycles 11-30 is pretty flat.

We would like to clarify that our slope-extraction approach shares a similar underlying logic with the selection process used in HRR, in both cases, the reported flux is identified by locating the region of the recording where the respiration is strong and stable, rather than by averaging across the entire measurement period for a given trait. In HRR, the operator visually identifies the plateau (strong and stable respiration) following substrate or inhibitor addition once the signal has stabilised. Our sliding-window approach identifies the analogous stable region in a consistent and replicable manner (programmable), by scanning for the window of consecutive time points that best reflects the established flux rather than transient noise. Notably, this window length is not fixed even in HRR. Oroboros's own DatLab guidance recommends a default slope-smoothing interval of 40–80 seconds for typical experiments, but explicitly extends this to 10 minutes or more when the signal is noisy, and beyond 30 minutes when flux is still rising toward a final steady state (Oroboros Instruments, Bioblast: "Flux analysis – DatLab"). Our ~6-minute sliding window falls within this same range, and reflects the same underlying principle: extending the window as needed to capture a genuinely stable signal.

To help researchers adopt the proposed approach, we provide supplementary materials that include a script for data extraction, visualisation, and analysis. We also explicitly state in the Discussion that: “Further protocol optimisation for each study system could focus on several aspects of the assay's data extraction and preparation to improve measures from this plate-reader approach, especially for lower-flux traits such as LEAK, which is slower and of smaller magnitude than OXPHOS” (Lines 718-721). As with high-resolution respirometry (HRR) measurements using the HHR Oroboros, we expect users to adjust the number of measurement cycles used in their analyses according to the respiratory characteristics of their tissue and study species. Our supplementary materials should help users process their data efficiently and make informed, study-specific decisions about the most appropriate number of cycles to include in their analyses.

References

Oroboros Instruments. Flux analysis – DatLab. Bioblast.
https://wiki.oroboros.at/index.php/Flux_analysis_-_DatLab

5. I appreciate the authors including information about the seahorse but they are still omitting the basic clark-type electrode means of measuring mito function. The oroboros is basically a very fancy version of this but the old-school electrode approach to measuring O2 consumption

© 2026. Published by The Company of Biologists under the terms of the Creative Commons Attribution License (https://creativecommons.org/licenses/by/4.0/).

from mitos is very cost-effective and requires minimal specialized equipment. I would argue this approach is cheaper than the plate-spec assay. Thus, the major advantage of the present assay is speed of throughput and NOT cost or availability of equipment.

We agree this is a fair omission to correct. We have added a discussion of Clark-type oxygen electrodes to the Introduction alongside our discussion of the Oroboros, noting that this approach is well established, requires comparatively minimal and inexpensive equipment, and predates chamber-based HRR systems such as the Oroboros (Lines 74–86).

We also agree that the plate reader is not the cheapest available option for measuring mitochondrial oxygen consumption, and we have revised our framing of cost-effectiveness throughout the manuscript accordingly (Lines 616–617 and Lines 621–624) so that we no longer imply cost alone as a distinguishing advantage. As the reviewer notes, Clark-type electrodes are cheaper than the plate reader, but like HRR they measure one sample at a time, and their comparatively lower sensitivity typically requires a higher mitochondrial density or respiratory flux to obtain a reliable signal (Silva & Oliveira, 2012). The plate reader's advantage over both existing options is not cost in isolation, but the combination of relatively low cost with high throughput: to our knowledge, it is the only current approach for measuring mitochondrial respiration that offers both. We have revised the relevant text to make this comparison explicit rather than presenting cost as a standalone selling point.

References

Silva & Oliveira (2012). *Methods in Molecular Biology*. https://doi.org/10.1007/978-1-61779-382-0_2

Reviewer 2:

The revised version of the manuscript, "High-throughput mitochondrial aerobic metabolism measurement: validating a plate-reader approach using avian blood cells", meets all the necessary changes asked by the reviwers. I appreciate the authors' effort and time for considering the suggestions and making the modifications. To my understanding this manuscript will add a valueable piece in the field of mitochondrial physiology and bioenergetics. I enjoyed reading it and am looking forward for it to be published.

We thank the reviewer for their positive assessment and for confirming that our revisions addressed their concerns from the previous round.

Third decision letter

MS ID#: bio.062712R2

MS Title: High-throughput mitochondrial aerobic metabolism measurement: assessing a plate-reader approach using avian blood cells

Authors: Cloé Hadjadji; Molly Ohse; Antoine Stier; Pierre Bize

I have been very impressed with the author's professionalism in responding to the main concerns and their dedication to additional validation experiments that were critically evaluated by the reviewer. I see both sides of the argument that the reviewer and authors provided, and the authors did well to address as many of the concerns as possible and spelled out the limitations for further development. Indeed, techniques can always be improved, but that is science. No single paper will have the best method.

© 2026. Published by The Company of Biologists under the terms of the Creative Commons Attribution License (https://creativecommons.org/licenses/by/4.0/).

That being said, I am happy to tell you that your manuscript has been accepted for publication in Biology Open, pending our standard publication integrity checks. It was accepted on 21st September 2026.

© 2026. Published by The Company of Biologists under the terms of the Creative Commons Attribution License (https://creativecommons.org/licenses/by/4.0/).
